# Seroprevalence of hepatitis B virus surface antigen (HBsAg) in Egypt (2000–2022): a systematic review with meta-analysis

**DOI:** 10.1186/s12879-023-08110-5

**Published:** 2023-03-10

**Authors:** Ahmed Azzam, Heba Khaled, Ola A. Elbohy, Shueb Abdirahman Mohamed, Sana Mostafa Hussein Mohamed, Ahmed H. Abdelkader, Ahmad Ashraf Ezzat, Amora Omar Ibrahim Elmowafy, Ola Ali El-Emam, Mona Awadalla, Neveen Refaey, Shimaa Mohamed Abdou Rizk

**Affiliations:** 1 Department of Microbiology and Immunology, Faculty of Pharmacy, Helwan University, Ain Helwan, Cairo, Egypt; 2 Department of Biochemistry, Faculty of Pharmacy, Cairo University, Cairo, Egypt; 3 Department of Virology, Faculty of Veterinary Medicine, Mansoura University, Mansoura, 35516 Egypt; 4 Faculty of Medicine, Alexandria University, Alexandria, Egypt; 5 Department of Oral Biology, Faculty of Dentistry, Cairo University, Cairo, Egypt; 6 Department of Microbiology Faculty of Veterinary Medicine, Cairo University, Cairo, Egypt; 7 Faculty of Pharmacy, Minia University, Minya, Egypt; 8 Department of Medical Surgical Nursing-Faculty of Nursing, Mansoura University, Mansoura, Egypt; 9 Department of Clinical Pathology, Faculty of Medicine, Mansoura University, Mansoura, Egypt; 10 Department of Oral Surgery, Faculty of Dentistry, Alexandria University, Alexandria, Egypt; 11 Department of Physical Therapy for Women’s Health, Faculty of Physical Therapy, Cairo University, Cairo, Egypt

**Keywords:** Epidemiology, Seroprevalence, Hepatitis B, HBV, Hepatitis B surface antigen, Egypt, Meta‑analysis

## Abstract

**Background:**

Hepatitis B infection seriously threatens global public health, especially in developing nations. Despite several investigations on HBV incidence, the national pooled prevalence remains unknown, particularly in populations at-risk at whom interventions should be primarily aimed.

**Methods:**

A comprehensive literature search of the following databases: Medline [PubMed], Scopus, Google Scholar, and Web of Science was conducted following the PRISMA guidelines. I-squared and Cochran's Q were used to measure the heterogeneity between the studies. Publications that matched the following were included: Primary studies published in Egypt from 2000 to 2022 reported HBV prevalence based on HBsAg. We excluded any studies that were not performed on Egyptians or that were performed on patients suspected of acute viral hepatitis or studies focusing on occult hepatitis or vaccination evaluation studies, or national surveys.

**Results:**

The systematic review included 68 eligible studies reporting a total of 82 incidences of HBV infection based on hepatitis B surface antigen with a total sample size of 862,037. The pooled national prevalence among studies was estimated to be 3.67% [95% CI; 3: 4.39]. Children under 20 with a history of HBV vaccination during infancy had the lowest prevalence of 0.69%. The pooled prevalence of HBV infection among pregnant women, blood donors, and healthcare workers was 2.95%, 1.8%, and 1.1%, respectively. While patients with hemolytic anemia and hemodialysis patients, patients with malignancies, HCC patients, and chronic liver disease patients had the highest prevalences at 6.34%, 25.5%, 18.6%, and 34%, respectively. Studies reporting HBV prevalence in urban settings compared to rural settings revealed a similar HBV prevalence of 2.43% and 2.15%, respectively. Studies comparing HBV prevalence in males and females revealed a higher prevalence among males (3.75%) than females (2.2%).

**Conclusion:**

In Egypt, hepatitis B infection is a significant public health issue. The blocking of mother-to-infant hepatitis B transmission, the scaling up of the scope of the existing vaccination program, and implementing new strategies, including screen-and-treat, may reduce the prevalence of the disease.

**Supplementary Information:**

The online version contains supplementary material available at 10.1186/s12879-023-08110-5.

## Background

Hepatitis B virus (HBV) is a partially double-stranded DNA virus belonging to the genus Orthohepadnavirus and the virus family Hepadnaviridae [1]. HBV specifically attacks the liver and can cause both acute and chronic diseases. The HBV life cycle is unique in that the circular partially double-stranded DNA (rcDNA) is converted to covalently closed circular DNA (cccDNA). The latter is used as a transcriptional template for all viral gene products, including the pregenomic RNA (pgRNA) [2]. Because current antiviral therapy with nucleos(t)ide analogues interferes with pgRNA reverse transcription and has limited effect on cccDNA, which appears as a stable minichromosome inside the nucleus of infected hepatocytes, HBV-infected patients require lifelong antiviral therapy [3–5].

There are 2 modes of HBV transmission: first, perinatal transmission or vertical transmission (from mother to child at birth). Second, horizontal transmission (transmission among individuals of the same generation). The most common mode of transmission changes with the endemicity of HBV. In areas with high endemicity, HBV is primarily transferred vertically from infected mothers to neonates around the time of birth; in addition, "horizontal" transmission by close contact between children has also been documented [6]. In low-endemicity areas, however, HBV infection is primarily acquired during adolescence and early adulthood and is strongly linked to high-risk behaviors such as unprotected sex and injectable drug use [6]. Chronic hepatitis B Antiviral therapy has decreased the rates of liver decompensation and, as a result, lowered hospitalization and mortality rates. Moreover, the longer-term benefits of antiviral therapy may include reversing liver fibrosis, reducing the risk of developing hepatocellular carcinoma, and decreasing the number of patients requiring liver transplantation [7–9].

Globally, an estimated 257–400 million people have chronic HBV infection [10]–[12]. Correspondingly, an estimated 29% of cirrhosis-related deaths worldwide were due to HBV [13]. Hepatitis B now ranks as the 15th leading cause of global mortality worldwide [14].

The prevalence of HBV varies worldwide, with the highest levels in sub-Saharan Africa and some countries in the Western Pacific region [10, 13]. An earlier meta-analysis conducted in Egypt that included 13 studies covering the period from 1983 to 2002 estimated the pooled prevalence of HBV to be 6.7% among healthy populations and 25.9% among hepatocellular carcinoma (HCC) [15]. The most recent Egyptian Health Issues Survey (EHIS), conducted in 2015 by El-Zanaty and colleagues, estimated a 1% prevalence of HBV infection based on HBsAg seroprevalence among 26,047 healthy participants aged 1–59 years and a 1.56% among 16,003 healthy participants aged 15–59 years [16].

Despite several investigations examining the incidence of HBV, the national pooled prevalence of HBV in Egypt remains unknown, especially in specific subpopulations at which intervention should be aimed. So we conducted a systematic review with meta-analysis to overcome the shortcomings of individual research and promote an improved understanding of HBV epidemiology and provide the evidence necessary to guide research, policy, and programmatic efforts in Egypt.

## Material and methods

### Search strategy

A comprehensive literature search of the following databases: MEDLINE [PubMed], Scopus, Google Scholar, and Web of Science was conducted using the following keywords: hepatitis B, Hepatitis B virus (HBV), hepatitis B surface antigen (HBsAg), viral liver disease, viral hepatitis, and Egypt. The review was conducted following the PRISMA (Preferred Reporting Items for Systematic Reviews and Meta-Analyses) Statement [17] and was registered in the PROSPERO International prospective register of systematic reviews, registration number CRD42022338782.

Additional file 1: Tables S1 and S2 illustrate the preferred reporting items for systematic reviews and meta-analyses checklist and the search strategy used in PubMed, respectively.

### Inclusion and exclusion of studies

We included studies that fully satisfied all of the following: Only primary studies (cross-sectional, case–control, or cohort studies) of participants residing in Egypt, Studies reporting the prevalence of HBV infection based on the presence of hepatitis B surface antigen and published in English between January 1st, 2000, and August 1st, 2022.

Studies were excluded if any of the following conditions were met: studies focusing on occult infection studies that were not carried out in Egypt or on Egyptian immigrants, Patients suspected of having acute viral hepatitis, non-human studies, the HBsAg detection method was not clear, and the full text wasn't available. We also excluded national surveys, studies based on the data of a national survey, and vaccination evaluation studies.

Studies were selected based on the aforementioned inclusion and exclusion criteria by two independent authors (AAZ, HK). Any disagreement was settled by consensus among all authors.

### Data extraction

The data extraction was conducted by four investigators (AAZ, HK, NR, and MA) and cross-checked by the rest of the authors.

From each included study, the following was extracted: the last name of the first author; publication time; study period; sample size; total HBsAg positive patients; region, population; study; age range; male/female%; setting (urban/rural%); and participants with anti-HBs levels of less than 10 IU/L.

To decrease the heterogeneity between studies, we classify them based on the risk of exposure into:

(A) Low-risk populations are subdivided into children under the age of 20 with a history of HBV vaccination during childhood, healthy adults, pregnant women, and blood donors.
(B) Intermediate-risk populations, such as healthcare workers and other workers who may be at risk due to occupational exposure, such as barbers or waste and sewage workers.
(C) High-risk populations, such as patients with hemolytic anemia who require blood transfusions or patients with end-stage renal failure who require hemodialysis, as well as people who have had direct contact with HBV and HIV-infected patients.
(D) Patients with liver-related conditions.
(E) Patients with special conditions such as malignancies and other special cases.

### Quality assessment

The quality of the included studies was checked using a 12-point scoring system based on the Downs and Black checklist [18], adopted in similar reviews [19, 20] by three reviewers(OAE, SAM, and SMHM) and crosschecked by two independent reviewers (AAE, AHA). The 12-points were: (objective of the study was clearly described, the study design was clearly stated, participants were representative of the population from which they were recruited, participants accrued during the same time, modest sample size, management of missing data, age, gender and other characteristics explored/reported, e.g. were confounders reported, was detection method of HBV reported, were potential biases reported, was outcome clearly described?

Studies were classified into 3 grades: Grade A (12–9), Grade B (8–5), and Grade C (4–1).

### Data synthesis

I-squared and Cochran's Q were used to measure the heterogeneity between the studies and based on the random effects model, results were reported as proportions with a 95% confidence interval (CI). Analyses of the subgroups were conducted based on the aforementioned subclasses and demographics of the participants. All statistical analysis was performed using StatsDirect statistical software (Version 3.0.0, StatsDirect Ltd, Cheshire UK). Publication bias testing by funnel plot and associated tests were not conducted as they do not produce reliable results for meta-analysis of proportions [21].

## Results

### Study selection

**Fig. 1:**
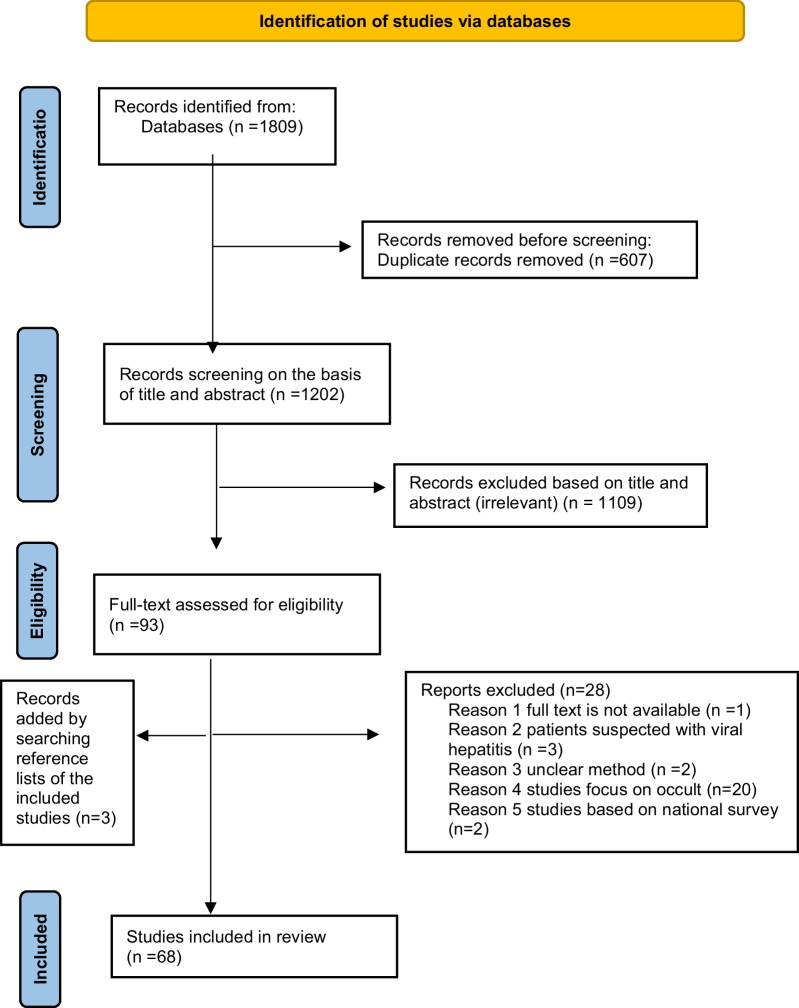
Flow chart depicting the selection of publications

Figure 1 outlines the schematic flow of the studies identification and inclusion processes. A total of 1809 records were identified through the literature search. 607 duplicates were removed. The remaining 1202 publications were then evaluated by title and abstract, and 1109 articles were found to be irrelevant and excluded. The remaining 93 articles were reviewed for eligibility using full text, and 28 were rejected. Three additional eligible studies were identified by searching the reference lists of the included studies, bringing the total number of studies included to 68 [22–89].

### The characteristics of the included studies

The characteristics of the studies included are shown in Additional file 1: Tables S3–S7. Fourteen (14) of the 68 included studies were published from 2000 to 2009, while 54 studies were published from 2010 to August 2022. Seventeen studies were graded as "B" and 51 studies as "A". 96.34% of the total sample size was for the low-risk population, 0.58% for intermediate, 0.2% for high-risk, 2.77% for patients with liver-related conditions, 0.067% for patients with malignancies, and 0.04% for patients with heterogeneous cases (Table 1).

**Table 1:** Meta-analysis of HBV prevalence among subgroups in Egypt

Group	Subgroup	No. of estimates	Sample size (n)	Pooled proportion (%)	95% CI (%)	Heterogeneity		
						I^2^% (inconsistency)	Cochran Q	P value
Low-risk	Children below 20 years with a history of HBV vaccination during infancy	8	5960	0.69	[0.23:1.4]	76.2	29.4	0.0001
	Healthy adults	12	68,494	2.4	[1.28: 3.9]	74.3	42.8	< 0.0001
	Pregnant women	9	5522	2.95	[1.6:4.6]	89.8	78.3	< 0.0001
	blood donors	15	750,515	1.8	[1.4:2.3]	99.4	2235	< 0.0001
	total	44	830,491	1.93	[1.55:2.35]	99	4452.6	< 0.0001
Intermediate-risk	Health care workers	7	2554	1.1	[0.55:1.8]	53.9	13	0.043
	Other workers	3	2502	2.73	[1.26:4.73]	83.9	12.4	0.002
	total	10	5056					
High-risk	Patients with hemolytic anemia and hemodialysis patients	10	1440	6.34	HBV-infected	95.8	216.3	< 0.0001
	direct contact with HBV-infected patients	1	154	–	–	–	–	–
	HIV-infected patient	1	141	–	–	–	–	–
	total	12	1735	5.86	[1.8:12]	95.2	230.4	< 0.0001
Liver related conditions	HCC	2	2362	18.6	[4.69:38.9]	99.2	125.6	< 0.0001
	HCV	2	308	1.57	[0.49:3.25]	0	0.257	0.6117
	CLD	2	21,215	34	[8.8:65.59]	97.5	40	< 0.0001
	Total		23,885					
Special cases	Patients with malignancies	6	526	25.5	[14.37:38.6]	89.5	47.6	< 0.0001
	Heterogeneous cases	4	345	2.27	[0.95:4.1]	2.7	3.08	0.3788
Overall total		82	862,037	3.67	[3:4.39]	99.5	15,617	< 0.0001

*HCC* Hepatocellular carcinoma, *HCV* hepatitis C virus, *CLD* chronic liver disease

### The pooled national prevalence

A total of 68 eligible reports were included in the systematic review, with an overall sample size of 862,037. Based on HBV surface antigen testing, 82 incidences of HBV infection were reported (Fig. 1). The pooled prevalence across studies, as shown in Fig. S1, was calculated to be 3.67% (95% CI: 3: 4.39), with a high degree of heterogeneity, as shown by I^2^ = 99.5%. The pooled prevalence of HBV infection with 95% CI for all categories is shown in Table 1, along with an evaluation of the heterogeneity.

### Sub-group analysis based on the population's risk

#### Low-risk population

**Fig. 2:**
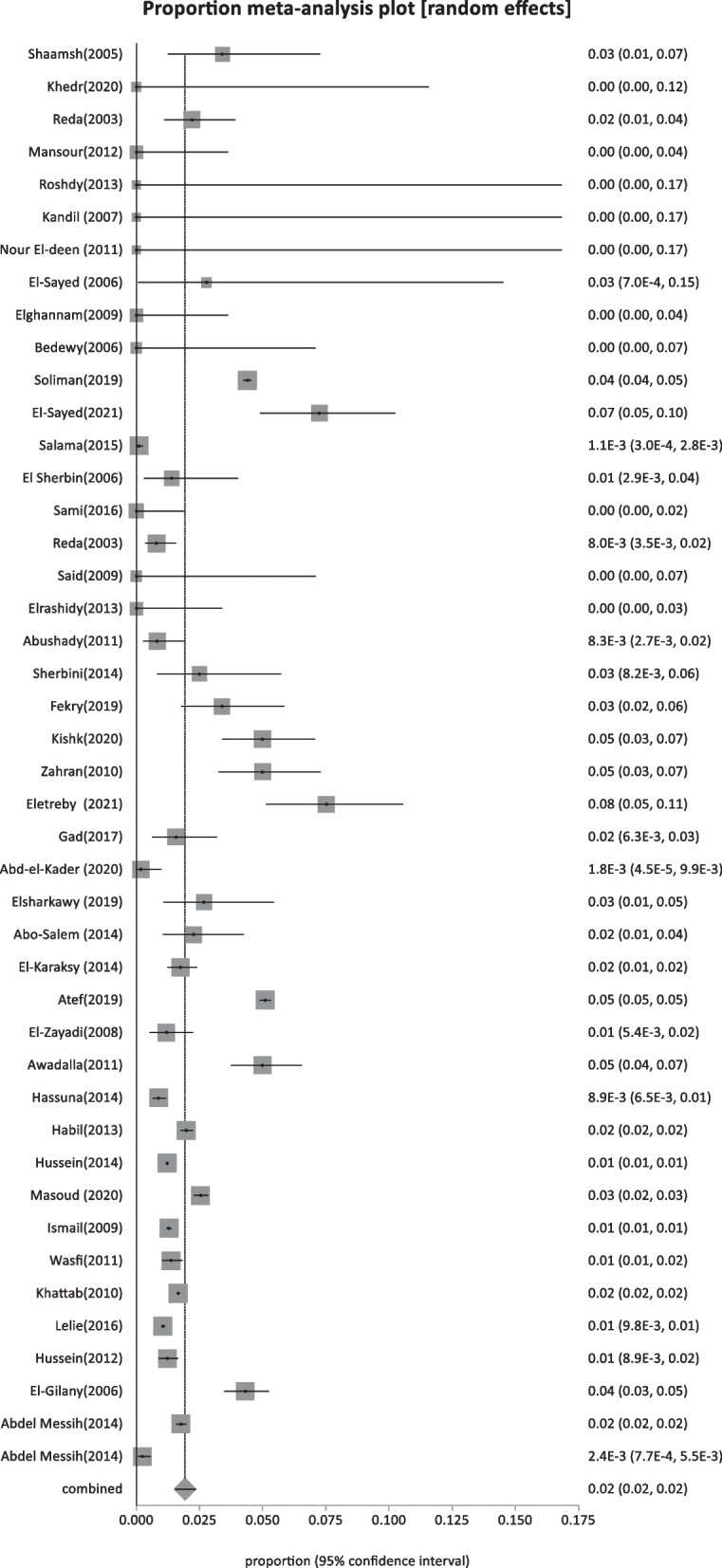
Forest plot of HBV seroprevalence among the low-risk population

**Fig. 3:**
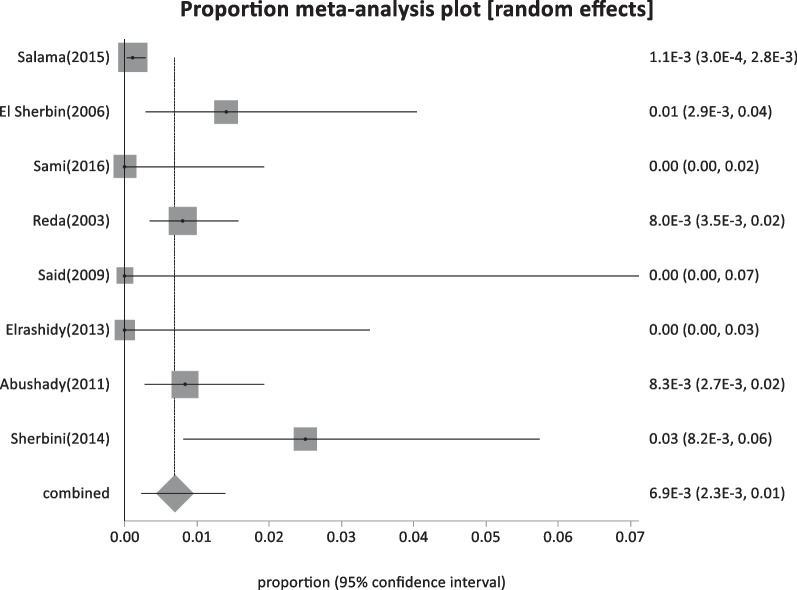
Forest plot of Children below 20 years of age with a history of HBV vaccination during infancy

**Fig. 4:**
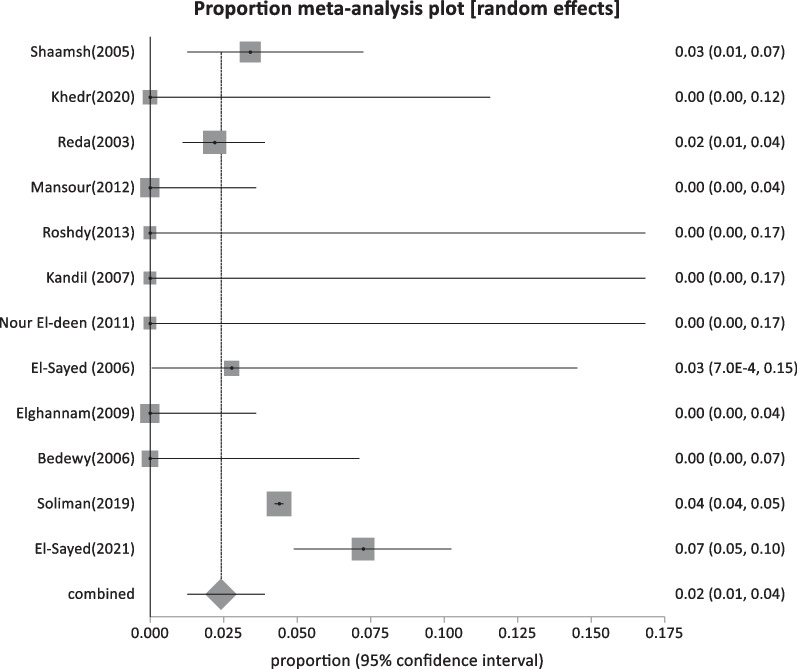
Forest plot of HBV among healthy adults

**Fig. 5:**
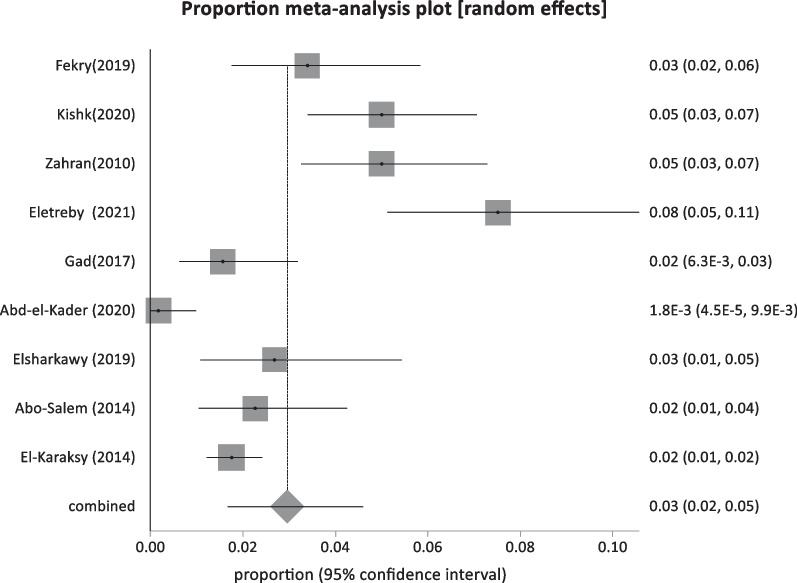
Forest plot of HBV among pregnant women

**Fig. 6:**
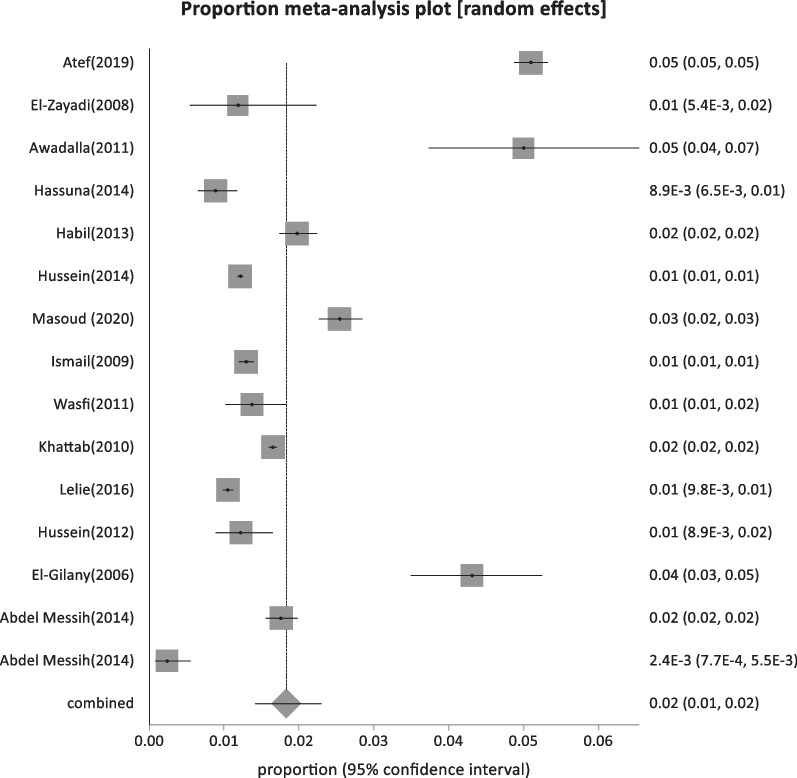
Forest plot of HBV among blood donors

Forty-four studies were classified as low-risk studies, totaling 830,491 people. The pooled prevalence of these studies was estimated to be 1.93% (95% CI: 1.55–2.35) (Fig. 2), with a high heterogeneity of 99% by I^2^%. Eight studies reported the prevalence of HBsAg in children below 20 years with a history of HBV vaccination during infancy (sample size: 5960) and revealed the lowest prevalence of 0.69% (95% CI; 0.23–1.4) (Fig. 3), with 76.2% heterogeneity based on I^2^%. Twelve studies reported the prevalence of HBsAg among healthy adults (sample size: 68,494) with a pooled prevalence of 2.4% (95% CI; 1.28: 3.9) (Fig. 4). Nine studies discussed the prevalence of HBV in pregnant women (sample size: 5522) and showed the highest prevalence of 2.95% (95% CI; 1.6–4.6) (Fig. 5), among the low-risk population but 95% CI overlapped. There were fifteen studies investigating HBsAg prevalence in blood donors with a sample size of 750,515; these studies had a pooled proportion of 1.8% (95% CI; 1.4: 2.3) (Fig. 6).

#### Intermediate-risk population

**Fig. 7:**
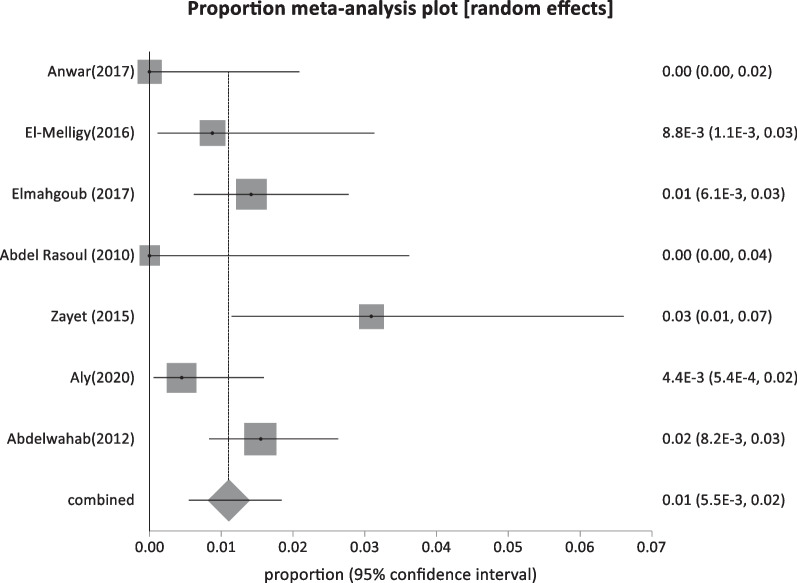
Forest plot of HBV seroprevalence among Health care workers

**Fig. 8:**
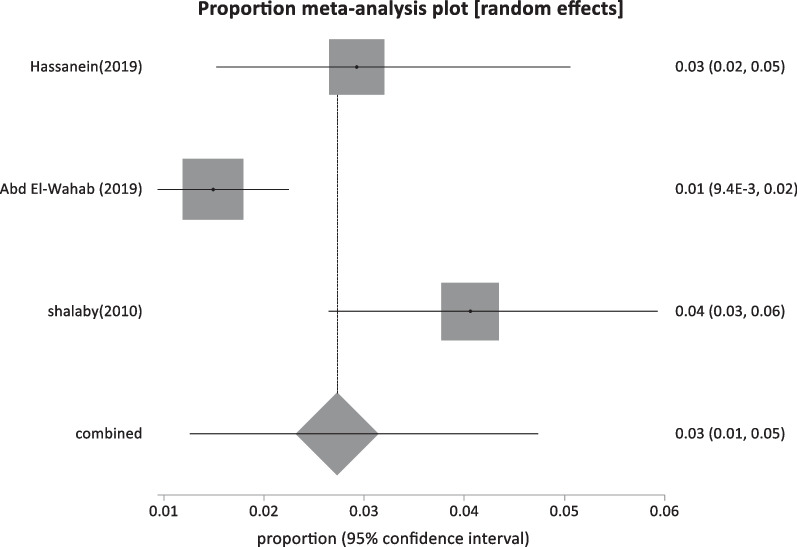
Forest plot of HBV seroprevalence among barbers, waste, and sewage workers

Ten studies were included in the intermediate-risk population group: seven reported HBsAg prevalence among healthcare workers, and three studies examined other occupationally exposed workers like barbers or waste and sewage workers. Unexpectedly, the pooled prevalence of HBV infection among healthcare workers was low at 1.1% (95% CI: 0.55:1.8), with a moderate level of heterogeneity (I^2^ = 53.9%) (Fig. 7) and a total sample size of 2554. Three studies with a total sample size of 2502 reporting HBsAg prevalence among barbers or waste and sewage workers revealed a pooled proportion of 2.73% (95% CI; 1.26:4.73) (Fig. 8), and a high level of heterogeneity (I^2^ = 83.9%).

#### High-risk population

**Fig. 9:**
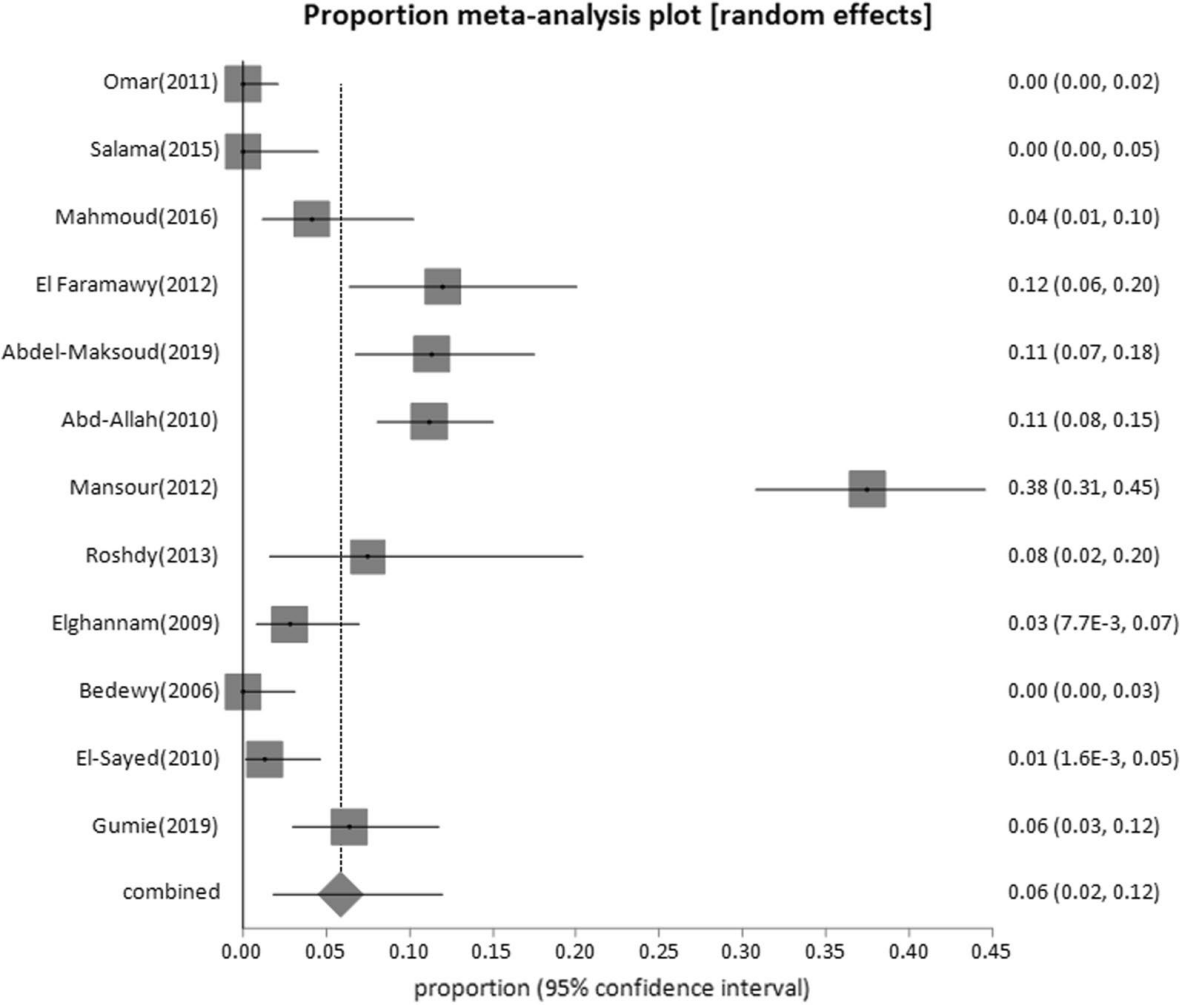
Forest plot of HBV seroprevalence among high-risk population

**Fig. 10:**
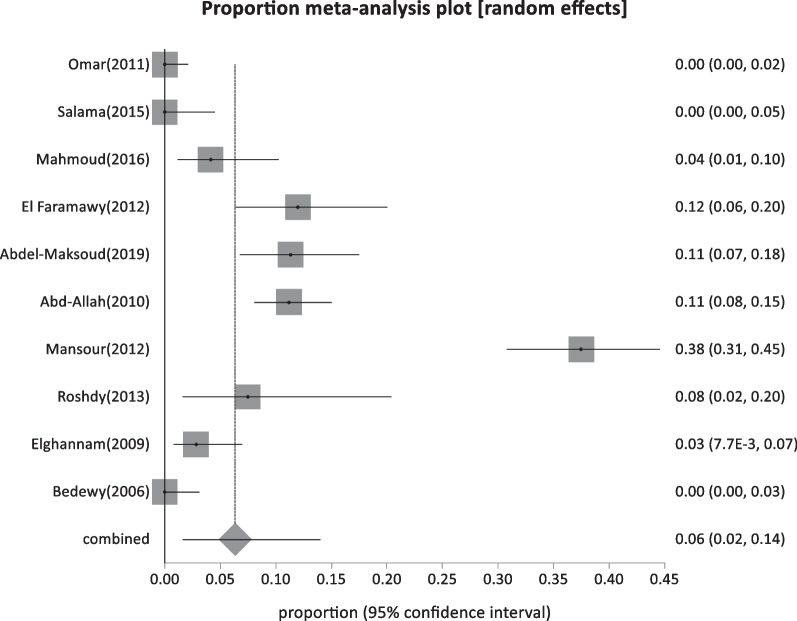
Forest plot of HBV seroprevalence among Patients with hemolytic anemia requiring blood transfusion or with end-stage renal failure requiring hemodialysis

The high-risk population group included twelve reports: ten studies on patients with hemolytic anemia requiring blood transfusion or with end-stage renal failure requiring hemodialysis (sample size: 1440), one study on individuals with direct contact with HBV-infected patients (sample size: 154), and one study on HIV-infected patients (sample size: 141), with a total sample size of 1735 (Table 1). As represented in Table 1, the pooled prevalence among the high-risk population was identified at 5.86% (95% CI; 1.8:12), while it was 6.34% (95% CI; 1.56:14) in patients with hemolytic anemia and the hemodialysis group (Figs. 9, 10, respectively).

#### Patients with liver-related conditions

**Fig. 11:**
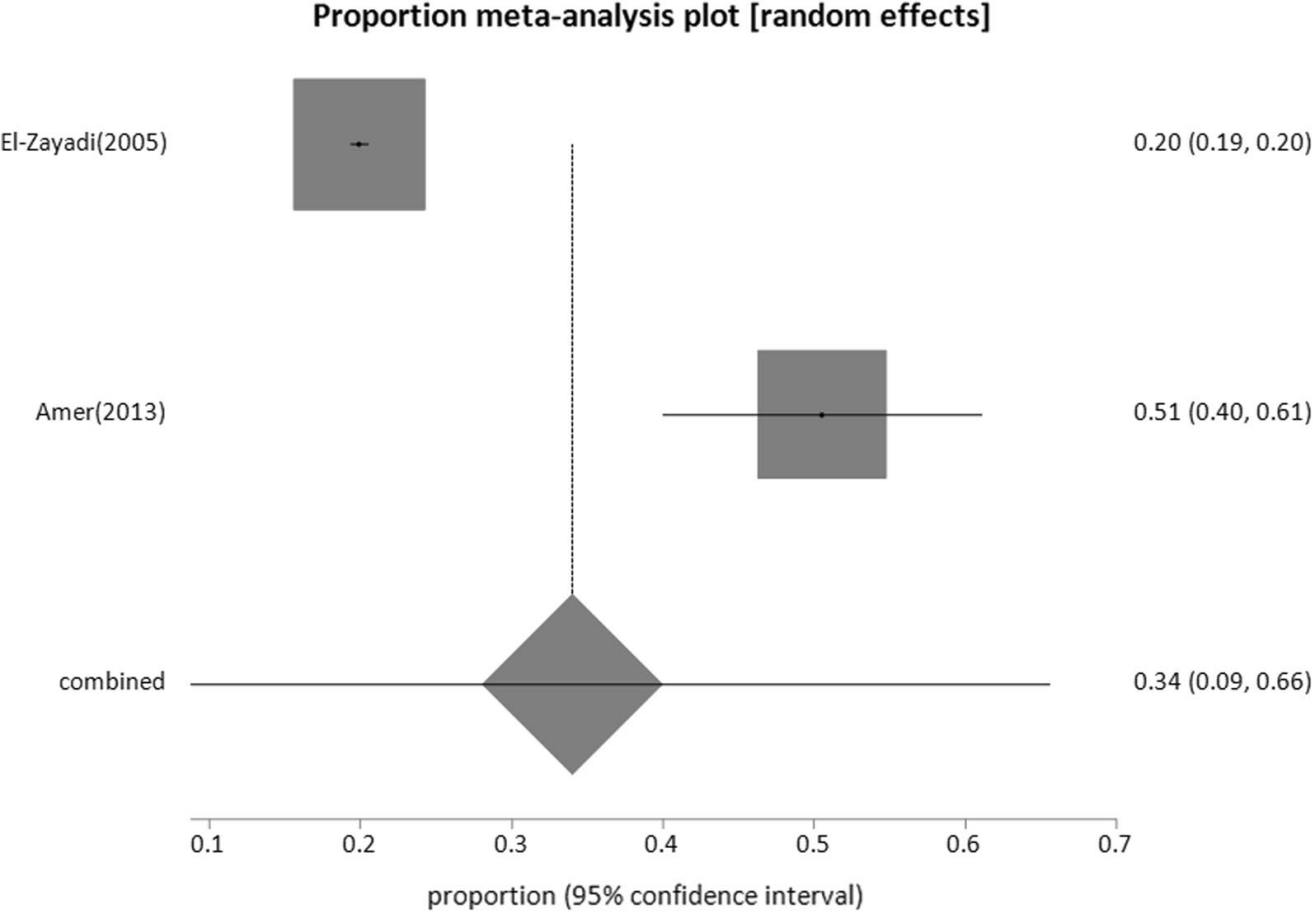
Forest plot of HBV prevalence among patients with chronic liver disease

**Fig. 12:**
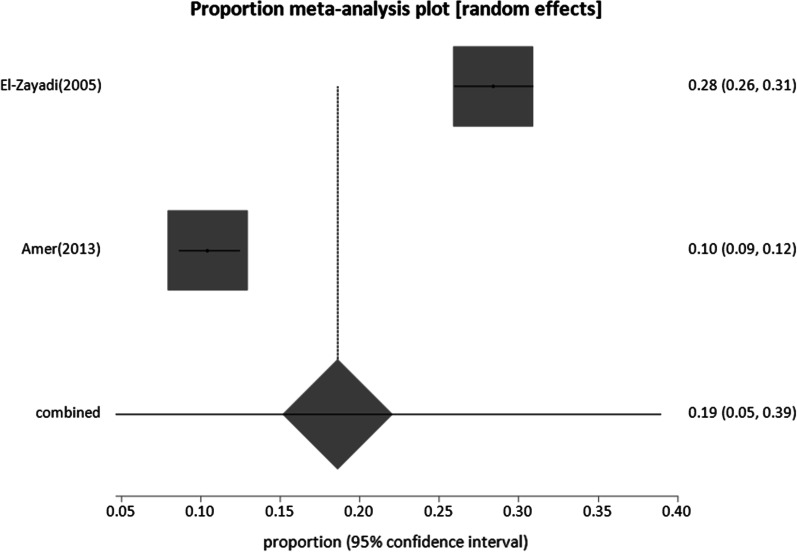
Forest plot of HBV seroprevalence among Patients HCC

**Fig. 13:**
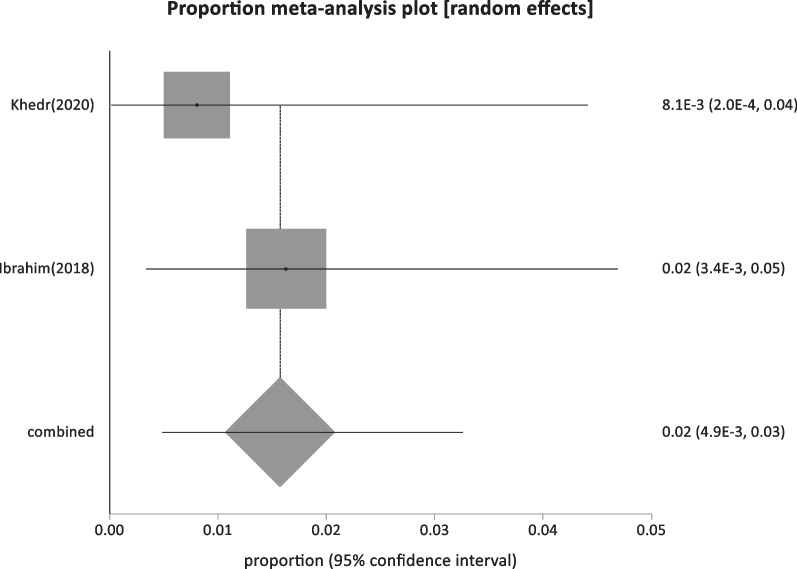
Forest plot of HBV seroprevalence among Patients with HCV

Four studies reporting six incidences of HBV infection, two on HCC, two on HCV, and two on patients with chronic liver diseases were included. The total sample size for these studies was 23,885. Patients with chronic liver disease had the highest prevalence of 34% (95% CI; 8.8:65.59), followed by HCC patients with 18.6% (95% CI; 4.69:38.9), while the prevalence was the lowest in HCV-infected patients at 1.57% (95% CI; 0.49:3.25) (Figs. 11, 12, 13, respectively).

#### Special clinical cases

**Fig. 14:**
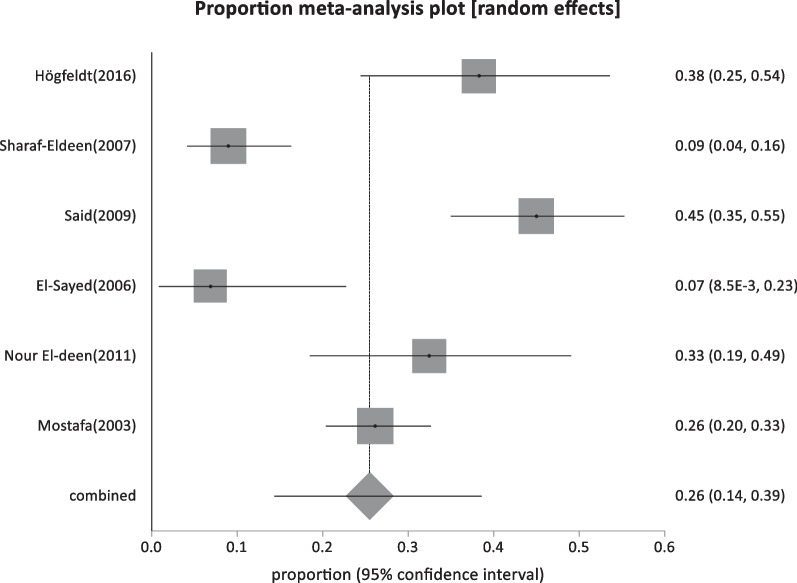
Forest plot of HBV seroprevalence among Patients With malignancies

Six studies reported HBsAg prevalence in patients with malignancies with a pooled proportion of 25.5% (95% CI; 14.37:38.6) and a total sample size of 526 (Table 1; Fig. 14).

**Fig. 15:**
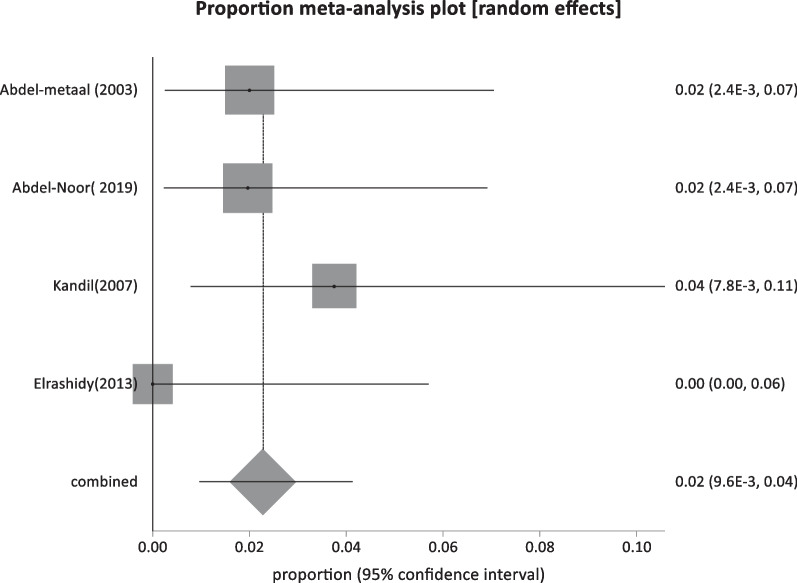
Forest plot of HBV seroprevalence among patients with heterogeneous clinical cases

Another four studies reported HBsAg prevalence in patients with rheumatoid arthritis and diabetes mellitus, with a total sample size of 345 and a pooled prevalence of 2.27% (95% CI: 0.95: 4.1) (Fig. 15).

### Sub-group analyses based on gender and setting

Nine studies reporting HBV prevalence among male participants compared to female participants revealed a higher prevalence among males than females at 3.75% and 2.2%, respectively (Table 2) (Additional file 1: Figs. S2, S3). While eight studies comparing HBV prevalence in urban and rural settings found nearly similar HBV prevalence rates of 2.43% and 2.15%, respectively (Table 2) (Additional file 1: Figs. S4, S5, Tables S9, S10 present the characteristics of the studies according to gender and setting, respectively.

**Table 2:** Meta-analysis of HBV prevalence in Egypt according to gender and setting

	No. of estimates	Sample size (n)	Pooled proportion (%)	95% CI (%)	Heterogeneity		
					I^2^%	Cochran Q	P value
Male	9	49,523	3.75	[2.08:5.9]	98.5	532.3	< 0.0001
Female	9	38,796	2.2	[1.25:3.4]	87	61.7	< 0.0001
Rural	8	13,933	2.15	[1.18:3.4]	92	87.5	< 0.0001
Urban	8	8527	2.43	[1.45:3.65]	87.5	56.2	< 0.0001

## Discussion

Infection with the Hepatitis B virus (HBV) is a major threat to global public health, particularly in developing countries [90, 91]. Egypt has one of the highest HCV prevalences in the world [92]. In spite of this, there is still no accurate estimation of the level of HBV prevalence at the national level in Egypt. In 2015, the World Health Organization (WHO) set a target to eliminate hepatitis B by the year 2030. In compliance with this target, we performed a systematic review with meta-analysis to overcome the shortcomings of individual research and promote an improved understanding of HBV epidemiology, provide the evidence necessary to guide research, policy, and programmatic efforts in Egypt, and highlight the need for additional follow-up research and preventive measures in subpopulations with high HBV prevalence.

Levels of HBV endemicity based on HBsAg prevalence have been classified into four categories: low (2%), lower-intermediate (2–4.99%), higher-intermediate (5–7.99%), and high (≥ 8) [93]. Accordingly, the endemicity level of HBV infection in Egypt should be classified as lower-intermediate with a pooled prevalence of 3.67 (95% CI; 3: 4.39). A meta-analysis on the global prevalence of HBV infection done in the general population (blood donors, healthcare workers (HCWs), and pregnant women) estimated a 1.71% (95% CI; 1.67–1.76) HBsAg prevalence in Egypt [10]. However, that meta-analysis included studies from 1965 to 2013 without information on the source of data for each country. In addition, the review provided a pooled estimate of prevalence with no data on populations at-risk, at whom interventions should be primarily aimed. Similar studies found that rural areas had a higher prevalence of HBV than urban areas [94–97]. According to the current analysis, however, HBV prevalence was greater in urban than rural regions, at 2.43% and 2.15%, respectively, but their 95% confidence intervals overlapped, which is likely explained by an equally uninformed population on health and hygiene measures. Males were more likely than females to be infected with HBV (3.75% vs. 2.2%), but their 95% confidence intervals overlapped. This can be attributed to a tendency for Egyptian men to spend more time outdoors, making them more exposed to HBV infection.

The overall pooled HBV prevalence estimate among low-risk populations (1.93%) was significantly lower than previous meta-analysis of HBsAg prevalence in Egypt among healthy individuals (6.7%), which included studies from 1983 to 2002 [15]. Such a decrease in HBsAg prevalence may be attributed to the introduction of the HBV vaccination in 1992. The first step towards the eventual eradication of hepatitis B is the universal immunization of infants. In Egypt, the HBV vaccination program was applied in 1992 with a schedule of 2, 4, and 6 months of age [98]. Among all studied populations, children under 20 with a history of HBV vaccination in infancy had the lowest prevalence of 0.69%, indicating that HBV vaccination during infancy in Egypt provides adequate protection. The responsiveness to the HBV vaccine was also evaluated using the prevalence of unprotective levels of anti-HBs (< 10 IU/L) in a population with a history of infancy vaccination, which clearly demonstrated a high incidence of unprotective levels of anti-HBs over time, i.e., unprotective levels of anti-HBs were less common in children under 5 years and highest in those over 15 years (Additional file 1: Table S8). Long-term HBV vaccine protection should be investigated further in populations more than 20 years post-primary vaccination by assessing breakthrough infection and anti-HBs levels. The prevalence of HBV in pregnant females was the highest among the low-risk groups, with a pooled prevalence of 2.9%, but 95% CI overlapped. Hepatitis B is most commonly transmitted from mother to child during birth (vertical transmission) as well as horizontally during early childhood. These routes are also responsible for the vast majority of chronic infections [99]. Efforts should be coordinated to eliminate these transmission routes in Egypt as being the most important strategy to control the HBV epidemic. Pregnant women should be screened during their antenatal care, and newborns of infected mothers should be given hepatitis B immunoglobulin (HBIG). Transfusion of blood or blood products can result in the spread of infectious diseases when proper procedures are not followed. Our results revealed a relatively low prevalence of HBV infection among blood donors (1.8%), and this may be due to only healthy people aged 18–60 being allowed for blood donation.

A 2002 survey of Egyptian HCWs reported unsafe practices in the use and disposal of sharps and determined that HCWs had an average of 4.9 needlestick injuries per year [100]. Unexpectedly, the pooled prevalence of HBV infection among healthcare workers was low at 1.1% despite occupational exposure. This low prevalence may be explained by healthcare workers' growing understanding and awareness of infection control. HBV is stable on environmental surfaces for at least 7 days [101] therefore, it can be transmitted through accidental injuries to at-risk individuals as a result of their occupational exposure, e.g., sewage and waste workers and barbers and their clients. According to the three studies discussing this issue, HBV prevalence in barbers and their clients (4%) was higher than in waste workers and sewage workers (1.49% and 2.93%, respectively), suggesting a higher need for protection and follow-up for barbers.

Patients with hemolytic anemia and hemodialysis patients are especially vulnerable to infections, including HBV. According to the current analysis, the pooled HBV prevalence among those subpopulations is estimated at 6.34%. Strict adherence to standard infection prevention measures, regular screening of HBV markers, and, finally, HBV immunization for all patients may help minimize the incidence.

The high pooled estimates for HBsAg prevalence in patients with liver-related disorders, such as hepatocellular carcinoma and liver disease patients (Table 1) reflect the important role that HBV plays in the incidence of liver diseases in Egypt. Two studies found a prevalence of 1.57% of HBV among chronically infected patients with HCV (Additional file 1: Table S6). This may be explained by the ability of HCV to inhibit HBV replication, leading to a greater incidence of occult HBV (HBV DNA-positive and HBsAg-negative) [102, 103], which was not evaluated in the two studies and may have contributed to the low prevalence.

In addition to the patients' immunosuppressed states, patients with cancer frequently need several transfusions and are more likely to contract blood-transmissible diseases like HBV. This may explain the high incidence rate of HBV among patients with cancer (25.5%).

This current review provides the most updated figures regarding HBV prevalence and reflects the current situation in Egypt. However, there are several limitations to this review: First, studies used a variety of screening kits, and there may have been a difference in sensitivity and specificity between study periods, resulting in the different prevalence rates. Second, some studies had a small sample size. Third, not all studies reported the prevalence of HBV in rural compared to urban areas or males compared to females. Fourth, the overall prevalence may not be entirely representative of a true national prevalence, as there is no data about HBsAg prevalence in some regions.

## Conclusion

Our review highlights the prevalence of HBsAg in Egypt in the last two decades, particularly in those at high risk, for whom intervention should be targeted. More effort is needed to reduce infection rates by screening blood and blood products more thoroughly and emphasizing vaccination for those at high risk of infection. The universal immunization program, implemented in Egypt more than three decades ago, appeared to be effective. But universal antenatal hepatitis B virus screening programs also need to be implemented. Finally, community awareness will be required to properly address Egypt's HBV problem.

## Supplementary Information

**Additional file 1. Table S1**: Supplementary Preferred Reporting Items for Systematic Reviews and Meta-analyses (PRISMA) checklist. **Table S2**: PubMed search strategy for studies published between January 1st, 2000 and July 2022. **Table S3**: Studies reporting hepatitis B virus (HBV) seroprevalence among populations at low risk in Egypt. **Table S4**: Studies reporting hepatitis B virus (HBV) seroprevalence among populations at intermediate risk in Egypt. **Table S5**: Studies reporting hepatitis B virus (HBV) seroprevalence among populations at high risk in Egypt. **Table S6**: Studies reporting hepatitis B virus (HBV) seroprevalence patients with liver-related conditions. **Table S7**: Studies reporting hepatitis B virus (HBV) seroprevalence patients with specific clinical cases. **Table S8**: Prevalence of unprotective levels of anti-HBs (< 10 IU/L) in a population with a history of vaccination during infancy. **Table S9**: HBV prevalence according to gender. Table S10: HBV prevalence according to setting. **Fig. S1**: Forest plot of the prevalence of hepatitis B virus infection in all subpopulations in Egypt. **Fig. S2**: Forest plot of HBV prevalence among male participants. **Fig. S3**: Forest plot of HBV prevalence among female participants. **Fig. S4**: Forest plot of HBV prevalence in urban areas in Egypt. **Fig. S5**: Forest plot of HBV prevalence in rural areas in Egypt.

## Data Availability

All data generated or analyzed during this study are included in this published article [and its Additional file]. We have no conflicts of interest to disclose.

## Abbreviations

HBV: Hepatitis B virus
PRISMA: Preferred Reporting Items for Systematic Reviews and Meta-Analyses
HCC: Hepatocellular carcinoma
HIV: Human immunodeficiency virus
HBsAg: Hepatitis B surface antigen
HCV: Hepatitis C virus
HBIG: Hepatitis B immune globulin
HCWs: Healthcare workers
CLD: Chronic liver disease

## References

[1] Schaefer S (2007). Hepatitis B virus taxonomy and hepatitis B virus genotypes. World J Gastroenterol.

[2] Grimm D, Thimme R, Blum HE (2011). HBV life cycle and novel drug targets. Hepatol Int.

[3] Levrero M, Testoni B, Zoulim F (2016). HBV cure: why, how, when?. Curr Opin Virol.

[4] Ligat G, Goto K, Verrier E, Baumert TF (2020). Targeting viral cccDNA for cure of chronic hepatitis B. Curr Hepatol Reports.

[5] Allweiss L, Dandri M (2017). The role of cccDNA in HBV maintenance. Viruses.

[6] Trépo C, Chan HLY, Lok A (2014). Hepatitis B virus infection. Lancet.

[7] Peng CY, Chien RN, Liaw YF (2012). Hepatitis B virus-related decompensated liver cirrhosis: benefits of antiviral therapy. J Hepatol.

[8] Lu LG (2014). Antiviral therapy of liver cirrhosis related to hepatitis B virus infection. J Clin Transl Hepatol..

[9] Khoo T, Lam D, Olynyk JK (2021). Impact of modern antiviral therapy of chronic hepatitis B and C on clinical outcomes of liver disease. World J Gastroenterol.

[10] Schweitzer A, Horn J, Mikolajczyk RT, Krause G, Ott JJ (2015). Estimations of worldwide prevalence of chronic hepatitis B virus infection: a systematic review of data published between 1965 and 2013. Lancet.

[11] Revill PA, Chisari FV, Block JM, Dandri M, Gehring AJ, Guo H (2019). A global scientific strategy to cure hepatitis B. Lancet Gastroenterol Hepatol.

[12] Zuckerman JN, Zuckerman AJ (2000). Current topics in hepatitis B. J Infect.

[13] Hepatitis B. Accessed August 28, 2022. https://www.who.int/news-room/fact-sheets/detail/hepatitis-b.

[14] Lozano R, Naghavi M, Foreman K, Lim S, Shibuya K, Aboyans V (2012). Global and regional mortality from 235 causes of death for 20 age groups in 1990 and 2010: a systematic analysis for the Global Burden of Disease Study 2010. Lancet.

[15] Lehman EM, Wilson ML (2009). Epidemiology of hepatitis viruses among hepatocellular carcinoma cases and healthy people in Egypt: a systematic review and meta-analysis. Int J Cancer.

[16] Ministry of Health E, El-Zanaty and Associates, Egypt and ICF International (2015) Egypt Health Issues Survey 2015. Ministry of Health and ICF International, Cairo. Accessed August 28, 2022. https://dhsprogram.com/pubs/pdf/FR313/FR313.pdf.

[17] Moher D, Liberati A, Tetzlaff J, Altman DG (2009). Preferred reporting items for systematic reviews and meta-analyses: the PRISMA statement. BMJ.

[18] Downs SH, Black N (1998). The feasibility of creating a checklist for the assessing the methodological quality of both of randomised and non-randomised studies of health care interventions. J Epidemiol Community Heal.

[19] Ofori-Asenso R, Agyeman AA (2016). Hepatitis B in Ghana: a systematic review & meta-analysis of prevalence studies (1995–2015). BMC Infect Dis.

[20] Musa BM, Bussell S, Borodo MM, Samaila AA, Femi OL (2015). Prevalence of hepatitis B virus infection in Nigeria, 2000–2013: a systematic review and meta-analysis. Niger J Clin Pract.

[21] Hunter JP, Saratzis A, Sutton AJ, Boucher RH, Sayers RD, Bown MJ (2014). In meta-analyses of proportion studies, funnel plots were found to be an inaccurate method of assessing publication bias. J Clin Epidemiol.

[22] Gad MA, Metwally MA, Eissa HA, Gehad MA, Rayan MM (2017). Antenatal screening for hepatitis B virus infection. Benha Med J.

[23] Roshdy MN, Harfoush RA, Hamed NA, Morsi MG (2013). Quantitative estimation of interferon-gamma levels among Egyptian polytransfused haematology cases. East Mediterr Health J..

[24] El-Sayed GM, Mohamed WSE din, Nouh MA, Moneer MM, El-Mahallawy HA. Viral genomes and antigen detection of hepatitis B and C viruses in involved lymph nodes of Egyptian non-Hodgkin’s lymphoma patients. The Egyptian Journal of immunology/Egyptian Association of Immunologists. Published 2006. Accessed August 28, 2022. https://pubmed.ncbi.nlm.nih.gov/17974155/. 17974155

[25] Mansour AK, Aly RM, Abdelrazek SY, Elghannam DM, Abdelaziz SM, Shahine DA (2012). Prevalence of HBV and HCV infection among multi-transfused Egyptian thalassemic patients. Hematol Oncol Stem Cell Ther.

[26] Salama II, Sami SM, Said ZN, El-Sayed MH, El Etreby LA, Rabah TM (2015). Effectiveness of hepatitis B virus vaccination program in Egypt: multicenter national project. World J Hepatol.

[27] Ibrahim H, Ghaffar F, Shaer R, Madian M (2018). Prevalence of Epstein-Barr virus and hepatitis B virus infections among chronic HCV patients attending Kafer El Shiekh Liver and Heart Institute. Egypt J Adv Med Med Res.

[28] Reda AA, Arafa MA, Youssry AA, Wandan EH, Ab de Ati M, Daebees H (2003). Epidemiologic evaluation of the immunity against hepatitis B in Alexandria, Egypt. Eur J Epidemiol.

[29] Shalaby S, Kabbash IA, El Saleet G, Mansour N, Omar A, El Nawawy A (2010). Hepatitis B and C viral infection: prevalence, knowledge, attitude and practice among barbers and clients in Gharbia governorate, Egypt. East Mediterr Health J.

[30] El-Karaksy HM, Mohsen LM, Saleh DA, Hamdy MS, Yassin NA, Farouk M (2014). Applicability and efficacy of a model for prevention of perinatal transmission of hepatitis B virus infection: single center study in Egypt. World J Gastroenterol.

[31] Zahran KM, Badary MS, Agban MN, Abdel Aziz NHR (2010). Pattern of hepatitis virus infection among pregnant women and their newborns at the Women’s Health Center of Assiut University, Upper Egypt. Int J Gynaecol Obstet.

[32] Khattab MA, Eslam M, Sharwae MA, Hamdy L (2010). Seroprevalence of hepatitis C and B among blood donors in Egypt: Minya Governorate, 2000–2008. Am J Infect Control.

[33] Eletreby R, Elraouf MA, Fouad A, Nasser M, Al Bassiouni M, Zayed N, et al. Screening for chronic hepatitis C and chronic hepatitis B infections among pregnant females: a cross-sectional study. Egypt Liver J. 2021;11(1). 10.1186/s43066-021-00113-8.

[34] El-deen RAA, Harfoush RA, Elgharabawy MM, Hamed NA, Morsi MG (2011). Levels of interleukins 12 (IL-12) and 13 (IL-13), hepatitis B and C serology, and blood cultures among acute myeloid leukemia (AML) patients in Egypt. J Venom Anim Toxins Incl Trop Dis.

[35] Atef DM, Atef RM (2019). Usefulness of nucleic acid testing among negative HBs Ag blood donors in Egypt. Transfus Apher Sci.

[36] El-Zayadi AR, Ibrahim EH, Badran HM, Saeid A, Moneib NA, Shemis MA (2008). Anti-HBc screening in Egyptian blood donors reduces the risk of hepatitis B virus transmission. Transfus Med.

[37] Awadalla HI, Ragab MH, Osman MA, Nassar NA (2011). Risk factors of viral hepatitis B among Egyptian blood donors. Br J Med Med Res.

[38] Mahmoud RA, El-Mazary AAM, Khodeary A (2016). Seroprevalence of hepatitis C, hepatitis B, cytomegalovirus, and human immunodeficiency viruses in multitransfused thalassemic children in Upper Egypt. Adv Hematol..

[39] Said ZN, El-Sayed MH, El-Bishbishi IA, El-Fouhil DF, Abdel-Rheem SE, El-Abedin MZ (2009). High prevalence of occult hepatitis B in hepatitis C-infected Egyptian children with haematological disorders and malignancies. Liver Int.

[40] Khedr A (2020). Incidence and clinical implications of isolated hepatitis B core antibody serologic profile pattern among Egyptian patients with chronic hepatitis C. Egypt Acad J Biol Sci C Physiol Mol Biol..

[41] El-Melligy DM, Saad-Hussein A, Khalil SA (2016). Occupational exposure to hepatitis infection among Egyptian healthcare workers and hepatitis B vaccination. J Arab Soc Med Res.

[42] Zayet H, Ezz El-Din A, Ahmed S, El-Khayat M (2015). Hepatitis B and C virus infection among health care workers in General Surgery Department, Assiut University Hospitals, Egypt. J Occup Med..

[43] Elmaghloub R, Elbahrawy A, Didamony GE, Elwassief A, Saied Mohammad AG, Alashker A (2017). Hepatitis B virus genotype E infection among Egyptian health care workers. J Transl Intern Med.

[44] Abdel Rasoul G, ElBahnasy R, Michael A, Hendy O, Ahmed A (2010). Hepatitis B viral markers and vaccination status among health care providers in Menoufia Governorate. Egypt J Occup Med..

[45] Elghannam D, Aly R, Goda E, Eltoraby E, Farag R (2009). Clinical significance of antibody to hepatitis B core antigen in multitransfused hemodialysis patients. Asian J Transfus Sci.

[46] Hussein E, Teruya J (2012). Evaluation of blood supply operation and infectious disease markers in blood donors during the Egyptian revolution. Transfusion.

[47] Habil FE, Mahdi WKM, Abdelwahab SF, Abdel-Hamid M (2013). Hepatitis B virus genotype D predominates HBsAg-positive Egyptian blood donors and is mainly associated with a negative HBeAg serostatus. Intervirology.

[48] Amer NA, Gemaay MA, Mohamed AE, Hussein MM, Shehad I (2013). Prevalence of viral hepatitis in Egyptian patients with hepatocellular carcinoma. Egypt Liver J.

[49] Sharaf-Eldeen S, Salama K, Eldemerdash S, Hassan HMS, Semesem M (2007). Hepatitis B and C viruses in Egyptian children with malignancy. J Med Sci.

[50] Abdelkader AH, Ibrahim SA (2020). Prevalence of hepatitis B and C virus infection among pregnant women in Sharkia Governorate, Egypt. Afro-Egypt J Infect Endem Dis..

[51] Kandil ME, Rasheed MA, Saad NE (2007). Hepatitis C and B viruses among some high risk groups of Egyptian children. J Med Sci.

[52] Soliman G, Elzalabany M, Hassanein T, Miller D (2018). Mass screening for hepatitis B and C in South Upper Egypt: lessons learned from a real life experience. J Hepatol.

[53] Lelie N, Bruhn R, Busch M, Vermeulen M, Tsoi WC, Kleinman S, International NAT Study Group (2017). Detection of different categories of hepatitis B virus (HBV) infection in a multi-regional study comparing the clinical sensitivity of hepatitis B surface antigen and HBV-DNA testing. Transfusion.

[54] Abdel Messih IY, Ismail MA, Saad AA, Azer MR (2014). The degree of safety of family replacement donors versus voluntary non-remunerated donors in an Egyptian population: a comparative study. Blood Transfus.

[55] El Sherbini A, Mohsen SA, Seleem Z, Ghany AA, Moneib A, Abaza AH (2006). Hepatitis B virus among schoolchildren in an endemic area in Egypt over a decade: impact of hepatitis B vaccine. Am J Infect Control.

[56] Gamal Eldin Abdel-Metaal M, Abdulaal Doma M. Prevalence of hepatitis B and C in rheumatoid arthritis. AAMJ. 2003;1(2).

[57] Anwar MM, Ahmed D, Sheemy M, El-Tayeb M. Seroprevalence and risk factors for Hepatitis B and C among health care workers. Int J Infect Control. 2017;13(2). 10.3396/IJIC.V13I2.17459.

[58] Abo-Salem MES, Mahrous OAE, El-Shaarawy AA, Mohamed HM, Yehia SAS (2014). Seroprevalence of hepatitis B among pregnant women attending maternal and child health centres in Shebin El-Kom district (Menoufia governorate). Menoufia Med J.

[59] Gumie M, Saeed AB, Gad A, Abdelrahman AE, Elgayar A (2019). Prevalence of hepatitis C virus (HCV) and hepatitis B virus (HBV) co-infection among human immunodeficiency virus (HIV/AIDS). Egypt J Hosp Med..

[60] Shaamsh AH, Salem HT, Shaaban MM, Ghaneima SA, Helal SR (2005). Effect of uniplant on liver function in Egyptian women with asymptomatic hepatitis B virus infection. Afr J Reprod Health.

[61] Elrashidy H, Elbahrawy A, El-Didamony G, Mostafa M, George NM, Elwassief A (2013). Antibody levels against hepatitis B virus after hepatitis B vaccination in Egyptian diabetic children and adolescents. Hum Vaccines Immunother.

[62] El-Zayadi AR, Badran HM, Barakat EM, Attia Mel D, Shawky S, Mohamed MK, Selim O (2005). Hepatocellular carcinoma in Egypt: a single center study over a decade. World J Gastroenterol.

[63] Sami SM, Salama II, Abdel-Latif GA, El Etreby LA, Metwally AI, El Haliem NFA (2016). Hepatitis B seroprotection and the response to a challenging dose among vaccinated children in Red Sea Governorate. Open Access Maced J Med Sci.

[64] El-Faramawy AAM, El-Rashidy OF, Tawfik PH, Hussein GH (2012). Transfusion transmitted hepatitis: where do we stand now? A one center study in Upper Egypt. Hepat Mon.

[65] Hassanein F, Masoud I, Shehata A (2019). Infection hazard of exposure to intestinal parasites, *H. pylori* and hepatitis viruses among municipal sewage workers: a neglect high risk population. Parasitol United J..

[66] Elsharkawy SS, Elgazayerli WS, Elsharkawy SS, Elgazayerli WS (2017). Sero-prevalence of HBV, HCV and HEV among the Egyptian Pregnant Females. Open J Obstet Gynecol.

[67] Fekry MM, Hashish MH, Selim HS, Fawzy AM, Wahba MM (2019). Prevalence of hepatitis B virus among pregnant women attending antenatal care in Alexandria. J High Inst Public Health..

[68] El-Gilany AH, El-Fedawy S (2006). Bloodborne infections among student voluntary blood donors in Mansoura University, Egypt. Eastern Mediterr Health J.

[69] El-Sayed NA, Elshazly SH, Said ZN, El EAM, Abdelmageed NA (2021). Hepatitis B Seroprevalence among Egyptian University students in the postinfant compulsory vaccination period. Sci J Al-Azhar Med Fac Girls.

[70] Bedewy K, Yousry NI. TT-Virus and occult hepatitis B virus infections in Egyptian. Egypt J Med Microbiol. 2006;15(1).

[71] Ismail AM, Ziada HN, Sheashaa HA, Shehab El-Din AB (2009). Decline of viral hepatitis prevalence among asymptomatic Egyptian blood donors: a glimmer of hope. Eur J Intern Med.

[72] Manal Hamdy ES, Naglaa Ahmed A, Marwa Gomaa EF (2010). Intrafamilial transmission of hepatitis B and C among families of multi-transfused Egyptian children. Egypt J Community Med.

[73] Hassuna NA, Mohamed ZM, Abo-Eleuoon M, Abdel-Hamid M, Xu J (2015). Prevalence of hepatitis B virus (HBV), hepatitis C virus (HCV) infections and their co-infection among blood donors in Minia Governorate. Egypt J Adv Med Med Res.

[74] Högfeldt T, Jaing C, Loughlin KM, Thissen J, Gardner S, Bahnassy AA, Gharizadeh B (2016). Differential expression of viral agents in lymphoma tissues of patients with ABC diffuse large B-cell lymphoma from high and low endemic infectious disease regions. Oncol Lett.

[75] Abd-Allah E, Waked E, Assal HS, Younes K, Kantoush N (2010). Incidence of hepatitis C virus (HCV), hepatitis B virus (HBV) and dual infection in Egyptian patients on haemodialysis. Kidney.

[76] Abdel-Noor R, Watany M, Abd-Elsalam S, ElKhalawany W, Soliman S, Badawi R (2019). Is hepatitis B surface antigen (HB s Ag) enough alone as a screening test for HBV infection in rheumatic disease patients before starting immunosuppressive therapies? A cross-sectional study. Infect Disord Drug Targets.

[77] Aly H, Soliman N, Nemr N (2020). Hepatitis B virus sero-prevalence and vaccination status among health care workers, North East Egypt. Egypt J Med Microbiol..

[78] Salama K, Ibrahim O, Kaddah A (2015). Liver enzymes in children with beta-thalassemia major: correlation with iron overload and viral hepatitis. Artic Open Access Maced J Med Sci..

[79] Abd El-Wahab EW, Eassa SM (2019). Seroprevalence of HBV among Egyptian municipal solid waste workers. Heliyon..

[80] Kishk R, Mandour M, Elprince M, Salem A, Nemr N, Eida M (2020). Pattern and interpretation of hepatitis B virus markers among pregnant women in North East Egypt. Brazilian J Microbiol.

[81] Ahmed AMA, Temerk HA, Galal HR, Bazeed SES, Sultan S (2020). The seropervelance of infectious hepatitis viruses (HBV, HCV and HEV) among blood donors and their correlation to risk factors in Qena governorate. Upper Egypt VirusDisease.

[82] Hussein E (2014). Blood donor recruitment strategies and their impact on blood safety in Egypt. Transfus Apher Sci.

[83] Sherbini AS, Sherbiny HS (2014). Immunoprophylaxis of compulsory hepatitis B vaccination in Sharkia, Egypt. Afro-Egyptian J Infect Endem Dis..

[84] Wasfi OAS, Sadek NA (2011). Prevalence of hepatitis B surface antigen and hepatitis C virus antibodies among blood donors in Alexandria, Egypt. East Mediterr Health J.

[85] Abdel-Maksoud NHM, El-Shamy A, Fawzy M, Gomaa HHA, Eltarabilli MMA (2019). Hepatitis B variants among Egyptian patients undergoing hemodialysis. Microbiol Immunol.

[86] Omar N, Salama K, Adolf S, El-Saeed GSM, Abdel Ghaffar N, Ezzat N (2011). Major risk of blood transfusion in hemolytic anemia patients. Blood Coagul Fibrinolysis.

[87] Abdelwahab S, Rewisha E, Hashem M, Sobhy M, Galal I, Allam WR (2012). Risk factors for hepatitis C virus infection among Egyptian healthcare workers in a national liver diseases referral centre. Trans R Soc Trop Med Hyg.

[88] Mostafa A, Mansour T, Amin M, Khairy A, El Zomor H (2003). Seroprevalence of Hepatitis B and C in Pediatric Malignanices. J Egypt Nat Cancer Inst.

[89] Abushady EAE, Gameel MMA, Klena JD, Ahmed SF, Abdel-Wahab KSE, Fahmy SM (2011). HBV vaccine efficacy and detection and genotyping of vaccineé asymptomatic breakthrough HBV infection in Egypt. World J Hepatol.

[90] Hepatitis B (2010). Vaccines: WHO position paper-recommendations. Vaccine.

[91] GBD 2017 Causes of Death Collaborators (2015). Global, regional, and national age-sex specific all-cause and cause-specific mortality for 240 causes of death, 1990–2013: A systematic analysis for the Global Burden of Disease Study 2013. Lancet.

[92] Kouyoumjian SP, Chemaitelly H, Abu-Raddad LJ (2018). Characterizing hepatitis C virus epidemiology in Egypt: systematic reviews, meta-analyses, and meta-regressions. Sci Reports.

[93] Ott JJ, Stevens GA, Groeger J, Wiersma ST (2012). Global epidemiology of hepatitis B virus infection: new estimates of age-specific HBsAg seroprevalence and endemicity. Vaccine.

[94] Ajuwon BI, Yujuico I, Roper K, Richardson A, Sheel M, Lidbury BA (2021). Hepatitis B virus infection in Nigeria: a systematic review and meta-analysis of data published between 2010 and 2019. BMC Infect Dis.

[95] Bigna JJ, Amougou MA, Asangbeh SL, Kenne AM, Noumegni SRN, Ngo-Malabo ET (2017). Seroprevalence of hepatitis B virus infection in Cameroon: a systematic review and meta-analysis. BMJ Open.

[96] Wang H, Men P, Xiao Y, Gao P, Lv M, Yuan Q, et al. Hepatitis B infection in the general population of China: a systematic review and meta-analysis. BMC Infect Dis. 2019;19(1). 10.1186/S12879-019-4428-Y/FIGURES/4. 10.1186/s12879-019-4428-y PMC6751646 31533643

[97] Abesig J, Chen Y, Wang H, Mwekele F, Irene S, Wu XY (2020). Prevalence of viral hepatitis B in Ghana between 2015 and 2019: a systematic review and meta-analysis. PLoS ONE.

[98] Mansour E, Abdul-Rahim S, Batouty G, Zaghloul I, Abdel-Hadi S (1993). Integration of hepatitis B immunization in the Expanded Program on Immunization of the Child Survival Project. J Egypt Public Health Assoc.

[99] Hepatitis: preventing mother-to-child transmission of the hepatitis B virus. https://www.who.int/news-room/questions-and-answers/item/hepatitis-preventing-mother-to-child-transmission-of-the-hepatitis-b-virus. Accessed 4 Sept 2022.

[100] Talaat M, Kandeel A, El-Shoubary W, Bodenschatz C, Khairy I, Oun S (2003). Occupational exposure to needlestick injuries and hepatitis B vaccination coverage among health care workers in Egypt. Am J Infect Control.

[101] Bond WW, Favero MS, Petersen NJ, Gravelle CR, Ebert JW, Maynard JE (1981). Survival of hepatitis B virus after drying and storage for one week. Lancet (London, England).

[102] Yip TCF, Wong GLH (2019). Current knowledge of occult hepatitis B infection and clinical implications. Semin Liver Dis.

[103] Rodríguez-Iñigo E, Bartolomé J, Ortiz-Movilla N, Platero C, López-Alcorocho JM, Pardo M (2005). Hepatitis C virus (HCV) and hepatitis B virus (HBV) can coinfect the same hepatocyte in the liver of patients with chronic HCV and occult HBV infection. J Virol.

