# Supplementary material for: Seroprevalence of hepatitis B virus surface antigen (HBsAg) in Egypt (2000–2022): a systematic review with meta-analysis
Source: BMC Infect Dis. 2023 Mar 10;23:151. doi: 10.1186/s12879-023-08110-5 (PMC10007808; doi:10.1186/s12879-023-08110-5)
Supplement: Supplementary file 1 — Additional file 1. Table S1: Supplementary Preferred Reporting Items for Systematic Reviews and Meta-analyses (PRISMA) checklist. Table S2: PubMed search strategy for studies published between January 1st, 2000 and July 2022. Table S3: Studies reporting hepatitis B virus (HBV) seroprevalence among populations at low risk in Egypt. Table S4: Studies reporting hepatitis B virus (HBV) seroprevalence among populations at intermediate risk in Egypt. Table S5: Studies reporting hepatitis B virus (HBV) seroprevalence among populations at high risk in Egypt. Table S6: Studies reporting hepatitis B virus (HBV) seroprevalence patients with liver-related conditions. Table S7: Studies reporting hepatitis B virus (HBV) seroprevalence patients with specific clinical cases. Table S8: Prevalence of unprotective levels of anti-HBs (< 10 IU/L) in a population with a history of vaccination during infancy. Table S9: HBV prevalence according to gender. Table S10: HBV prevalence according to setting. Fig. S1: Forest plot of the prevalence of hepatitis B virus infection in all subpopulations in Egypt. Fig. S2: Forest plot of HBV prevalence among male participants. Fig. S3: Forest plot of HBV prevalence among female participants. Fig. S4: Forest plot of HBV prevalence in urban areas in Egypt. Fig. S5: Forest plot of HBV prevalence in rural areas in Egypt. [file 12879_2023_8110_MOESM1_ESM.docx]

**Additional file 1**

**Seroprevalence of hepatitis B surface antigen (HBsAg) in Egypt (2000–2022): a systematic review with meta-analysis**

Ahmed Azzam^1^, Heba Khaled^2^, Ola A. Elbohy^3^, Shueb Abdirahman Mohamed^4^, Sana Mostafa Hussein Mohamed^5^, Ahmed H. Abdelkader^6^, Ahmad Ashraf Ezzat^7^, Amora Omar Ibrahim Elmowafy^8^, Ola Ali El-Emam^9^, Mona Awadalla^10^, Neveen Refaey^11^, Shimaa Mohamed Abdou Rizk^8^

^1^ Department of Microbiology and Immunology, Faculty of Pharmacy, Helwan University, Cairo Egypt.
^2^ Department of biochemistry and molecular biology, Faculty of Pharmacy, Cairo University, Cairo, Egypt.
^3^ Department of Virology, Faculty of Veterinary Medicine, Mansoura University, Mansoura 35516, Egypt.
^4^ Faculty of Medicine, Alexandria University, Egypt.
^5^ Department of oral Biology, faculty of dentistry Cairo University, Egypt.
^6^ Department of Microbiology faculty of Veterinary Medicine Cairo University, Egypt.
^7^Faculty of pharmacy Minia University, Egypt.
^8^ Department of Medical Surgical Nursing-Faculty of Nursing -Mansoura University, Egypt.
^9^ Department of clinical pathology, Faculty of medicine, Mansoura University, Egypt.
^10^ Department of oral surgery, faculty of dentistry Alexandria University, Egypt.
^11^ Department of Physical Therapy for Women's Health, Faculty of Physical Therapy, Cairo University, Egypt.

**Contents:**

Table S1: Supplementary Preferred Reporting Items for Systematic Reviews and Meta-analyses (PRISMA) checklist

Table S2: PubMed search strategy for studies published between January 1st, 2000 and July 2022

Table S3: Studies reporting hepatitis B virus (HBV) seroprevalence among populations at low risk in Egypt

Table S4: Studies reporting hepatitis B virus (HBV) seroprevalence among populations at intermediate risk in Egypt

Table S5: Studies reporting hepatitis B virus (HBV) seroprevalence among populations at high risk in Egypt

Table S6: Studies reporting hepatitis B virus (HBV) seroprevalence patients with liver-related conditions

Table S7: Studies reporting hepatitis B virus (HBV) seroprevalence patients with specific clinical cases

Table S8: Prevalence of unprotective levels of anti-HBs (< 10 IU/L) in a population with a history of vaccination during infancy

Table S9: HBV prevalence according to gender

Table S10: HBV prevalence according to setting

Fig. S1: Forest plot of the prevalence of hepatitis B virus infection in all subpopulations in Egypt.

Fig. S2: Forest plot of HBV prevalence among male participants

Fig. S3: Forest plot of HBV prevalence among female participants

Fig. S4: Forest plot of HBV prevalence in urban areas in Egypt

Fig. S5: Forest plot of HBV prevalence in rural areas in Egypt

**Table S1: Supplementary Preferred Reporting Items for Systematic Reviews and Meta-analyses (PRISMA) checklist**

| **Section/topic** | **#** | **Checklist item** | **Reported on page #** |
| --- | --- | --- | --- |
| **TITLE** | | |  |
| Title | 1 | Identify the report as a systematic review, meta-analysis, or both. | 1 |
| **ABSTRACT** | | |  |
| Structured summary | 2 | Provide a structured summary including, as applicable: background; objectives; data sources; study eligibility criteria, participants, and interventions; study appraisal and synthesis methods; results; limitations; conclusions and implications of key findings; systematic review registration number. | 2&3 |
| **INTRODUCTION** | | |  |
| Rationale | 3 | Describe the rationale for the review in the context of what is already known. | 5 |
| Objectives | 4 | Provide an explicit statement of questions being addressed with reference to participants, interventions, comparisons, outcomes, and study design (PICOS). | 5 |
| **METHODS** | | |  |
| Protocol and registration | 5 | Indicate if a review protocol exists, if and where it can be accessed (e.g., Web address), and, if available, provide registration information including registration number. | 6 |
| Eligibility criteria | 6 | Specify study characteristics (e.g., PICOS, length of follow-up) and report characteristics (e.g., years considered, language, publication status) used as criteria for eligibility, giving rationale. | 6 |
| Information sources | 7 | Describe all information sources (e.g., databases with dates of coverage, contact with study authors to identify additional studies) in the search and date last searched. | 5&6 |
| Search | 8 | Present full electronic search strategy for at least one database, including any limits used, such that it could be repeated. | Table S.2 |
| Study selection | 9 | State the process for selecting studies (i.e., screening, eligibility, included in systematic review, and, if applicable, included in the meta-analysis). | 6 |
| Data collection process | 10 | Describe method of data extraction from reports (e.g., piloted forms, independently, in duplicate) and any processes for obtaining and confirming data from investigators. | 6&7 |
| Data items | 11 | List and define all variables for which data were sought (e.g., PICOS, funding sources) and any assumptions and simplifications made. | 7 |
| Risk of bias in individual studies | 12 | Describe methods used for assessing risk of bias of individual studies (including specification of whether this was done at the study or outcome level), and how this information is to be used in any data synthesis. | 7&8 |
| Summary measures | 13 | State the principal summary measures (e.g., risk ratio, difference in means). | 8 |
| Synthesis of results | 14 | Describe the methods of handling data and combining results of studies, if done, including measures of consistency (e.g., I^2^) for each meta-analysis. | 8 |

| Risk of bias across studies | 15 | Specify any assessment of risk of bias that may affect the cumulative evidence (e.g., publication bias, selective reporting within studies). | 8 |
| --- | --- | --- | --- |
| Additional analyses | 16 | Describe methods of additional analyses (e.g., sensitivity or subgroup analyses, meta-regression), if done, indicating which were pre-specified. | 8 |
| **RESULTS** | | |  |
| Study selection | 17 | Give numbers of studies screened, assessed for eligibility, and included in the review, with reasons for exclusions at each stage, ideally with a flow diagram. | 8&  Fig. 1 |
| Study characteristics | 18 | For each study, present characteristics for which data were extracted (e.g., study size, PICOS, follow-up period) and provide the citations. | Tables S3-S7 |
| Risk of bias within studies | 19 | Present data on risk of bias of each study and, if available, any outcome level assessment (see item 12). | Tables S3-S7 |
| Results of individual studies | 20 | For all outcomes considered (benefits or harms), present, for each study: (a) simple summary data for each intervention group (b) effect estimates and confidence intervals, ideally with a forest plot. | Fig.1-15 and Fig.S1-S5 |
| Synthesis of results | 21 | Present results of each meta-analysis done, including confidence intervals and measures of consistency. | Tables 1 & 2, Fig.1-15 and Fig.S1-S5 |
| Risk of bias across studies | 22 | Present results of any assessment of risk of bias across studies (see Item 15). | - |
| Additional analysis | 23 | Give results of additional analyses, if done (e.g., sensitivity or subgroup analyses, meta-regression [see Item 16]). | Tables 1 & 2 |
| **DISCUSSION** | | |  |
| Summary of evidence | 24 | Summarize the main findings including the strength of evidence for each main outcome; consider their relevance to key groups (e.g., healthcare providers, users, and policy makers). | 16-20 |
| Limitations | 25 | Discuss limitations at study and outcome level (e.g., risk of bias), and at review-level (e.g., incomplete retrieval of identified research, reporting bias). | 20 |
| Conclusions | 26 | Provide a general interpretation of the results in the context of other evidence, and implications for future research. | 20&21 |
| **FUNDING** | | |  |
| Funding | 27 | Describe sources of funding for the systematic review and other support (e.g., supply of data); role of funders for the systematic review. | 22 |

**Table** **S2: PubMed search strategy for studies published between January 1st, 2000 and August, 2022**

| **Search** | **Search terms** |
| --- | --- |
| **#1** | **((((hepatitis B) OR (Hepatitis B virus)) OR (HBV)) OR (viral liver disease)) OR (viral hepatitis)** |
| **#2** | **(hepatitis B surface antigen) OR (HBsAg)** |
| **#3** | **Egypt** |
| **#4** | **#1 AND #2** **AND #3** |
| **#5** | **Limit #4 in English and studies published between January 1st, 2000 and**  **July, 2022** |

**Table S3 Studies reporting hepatitis B virus (HBV) seroprevalence among populations at low risk in Egypt**

| **(A)Children below 20 years of age with a history of HBV vaccination during infancy (n=8)** | | | | | | | | | | | |
| --- | --- | --- | --- | --- | --- | --- | --- | --- | --- | --- | --- |
| **First author last name, year of**  **publication [citation]** | **Duration of sample collection** | **Sample**  **size** | **HBs Ag positive patients** | **region** | **population** | **Study design** | **Age *** | **M/F (%)** | **U/R (%)** | **Method** | **Quality** |
| **Salama(2015)**[1] | **July 2010 to June 2013** | **3600** | **4** | **Cairo,Beni-Sweif,Assiut-Dakahleya, Gharbeya and Red Sea)** | **healthy Children** | **cross-sectional** | **9M-16Y** | **48.4/51.6** | **53/47** | **ELISA** | **A** |
| **El Sherbin(2006)**[2] | **2002** | **214** | **3** | **Gharbeya** | **healthy children** | **Cross-sectional** | **6-10Y** | **N/P** | **N/P** | **Elisa** | **A** |
| **Sami(2016)**[3] | **July 2010 to June 2013** | **189** | **0** | **Red Sea** | **children** | **cross-sectional** | **9 M-16 Y** | **52.9/47.1** | **Mixed** | **ELISA** | **A** |
| **Reda(2003)**[4] | **N/P** | **1000** | **8** | **Alexandria** | **children** | **Case control** | **N/P** | **51.4/** **48.6** | **N/P** | **ELISA** | **A** |
| **Said(2009)**[5] | **6-month period** | **50** | **0** | **Cairo** | **healthy** | **case control** | **8.0 ± 2.1** | **54/46** | **N/P** | **ELISA** | **A** |
| **Elrashidy(2013)**[6] | **September 2012 to April 2013** | **107** | **0** | **Cairo** | **healthy** | **case control** | **5.5–15** | **45.8/54.2** | **N/P** | **ELISA** | **B** |
| **Abushady(2011)** [7] | **2004 to January 2007** | **600** | **5** | **Cairo/Menoufia /Qualyubia** | **healthy** | **cross-sectional** | **2-13** | **N/P** | **N/P** | **ELISA** | **B** |
| **Sherbini(2014)**[8] | **2011- 2013** | **200** | **5** | **Zagazig** | **apparently healthy Egyptian children** | **prospective comparative study** | **8M-12Y** | **N/P** | **N/P** | **ELISA** | **A** |
| **B: Adults (n=12)** | | | | | | | | | | |  |
| **Shaamsh(2005)**[9] | **N/P** | **176** | **6** | **Assiut** | **healthy women** | **cross-sectional** | **29.6±5.1** | **Female only** | **41.71/58.29** | **ELISA** | **A** |
| **Khedr(2020)**[10] | **N/P** | **30** | **0** | **N/P** | **Healthy adult** | **case control** | **31.061±10.093** | **83.3/16.7** | **N/P** | **ELISA** | **B** |
| **Reda(2003)**[4] | **N/P** | **500** | **11** | **Alexandria** | **unvaccinated children** | **Case control** | **N/P** | **54.6/45.4** | **N/P** | **ELISA** | **A** |
| **Mansour(2012)**[11] | **2009-2010** | **100** | **0** | **Mansoura** | **Healthy adult** | **case control** | **N/P** | **75/25** | **N/P** | **ELISA** | **A** |
| **Roshdy(2013)**[12] | **2010** | **20** | **0** | **Alexandria** | **Healthy adult** | **case control** | **20** | **N/P** | **N/P** | **ELISA** | **A** |
| **Kandil (2007)**[13] | **2004-2006** | **20** | **0** | **Cairo** | **healthy** | **case control** | **5-19** | **N/P** | **50/50** | **ELISA** | **A** |
| **Nour El-deen (2011)**[14] | **N/P** | **20** | **0** | **Alexandria** | **healthy** | **case control** | **N/P** | **N/P** | **N/P** | **ELISA** | **A** |
| **El-Sayed (2006)**[15] | **2002** | **36** | **1** | **Cairo** | **healthy** | **case control** | **3.5-67** | **N/P** | **N/P** | **ELISA** | **B** |
| **Elghannam(2009)**[16] | **N/P** | **100** | **0** | **Mansoura** | **healthy** | **case control** | **N/P** | **75/25** | **N/P** | **ELISA** | **A** |
| **Bedewy(2006)**[17] | **N/P** | **50** | **0** | **Beheira** | **healthy** | **case control** | **44.3 ± 13.3** | **62/38** | **N/P** | **ELISA** | **A** |
| **Soliman(2019)** [18] | **2016-2017** | **67042** | **2947** | **luxor** | **healthy** | **cross-sectional** | **43.6 ±14.3** | **47.7/52.3** | **N/P** | **ELISA** | **A** |
| **El-Sayed(2021)**[19] | **2015-2017** | **400** | **29** | **Cairo** | **healthy** | **cross-sectional** | **17-25** | **47/53** | **79/21** | **ELISA** | **A** |
| **C:Pregnant women (n=-9)** | | | | | | | | | | |  |
| **Fekry(2019)**[20] | **2016 - 2017** | **354** | **12** | **Alexandria** | **pregnant** | **cross-sectional** | **<30 -≥30** | **Female only** | **39.5/51.5** | **ELISA** | **A** |
| **Kishk(2020)**[21] | **-2018- 2019** | **600** | **30** | **Ismailia** | **pregnant** | **cross-sectional** | **18-45** | **Female only** | **32.2%/67.8** | **ELISA** | **A** |
| **Zahran(2010)**[22] | **2008 - 2009** | **500** | **25** | **Assiut** | **pregnant** | **cross-sectional** | **26.7±2.8** | **Female only** | **21.8%/78.2** | **ELISA** | **A** |
| **Eletreby (2021)**[23] | **Jan to Sep 2018** | **399** | **30** | **Cairo** | **pregnant** | **Cross-sectional** | **22-30** | **Female only** | **54.9/45.1** | **ELISA** | **A** |
| **Gad(2017)**[24] | **2013 -2014** | **448** | **7** | **Qualyubia** | **pregnant** | **cross-sectional study** | **19-44** | **Female only** | **N/P** | **ELISA** | **A** |
| **Abd El-Kader (2020)**[25] | **2017-2019** | **563** | **1** | **Zagazig** | **Pregnant** | **cross-sectional study** | **17-30 years (86.3%)** | **Female only** | **19.9/80.1** | **ELISA** | **A** |
| **Elsharkawy (2019)**[26] | **January to July 2017** | **261** | **7** | **Alexandria and Beheira** | **Pregnant** | **cross-sectional study** | **18-42** | **Female only** | **57.5/42.5** | **ELISA** | **A** |
| **Abo-Salem (2014)**[27] | **N/P** | **397** | **9** | **Menoufia** | **Pregnant** | **cross-sectional** | **N/P** | **Female only** | **N/P** | **ELISA** | **B** |
| **El-Karaksy (2014)**[28] | **May 2010 to July 2011** | **2000** | **35** | **Cairo** | **pregnant** | **prospective cohort** | **27.1 ± 4.8** | **Female only** | **N/P** | **rapid test (one step HBs Ag test)** | **A** |
| **D:Blood donors(n=15)** | | | | | | | | | | |  |
| **Atef(2019)**[29] | **2017 -2018** | **36534** | **1863** | **Zagazig** | **blood donors** | **cross-sectional** | **18 -56** | **94.3/5.7** | **N/P** | **chemoleucent- Assay** | **B** |
| **El-Zayadi(2008)**[30] | **2005** | **760** | **9** | **Cairo** | **blood donors** | **cross-sectional** | **18-54** | **83.7/16.3** | **N/P** | **ELISA** | **A** |
| **Awadalla(2011)**[31] | **N/P** | **1000** | **50** | **Cairo** | **healthy blood donors** | **cross-sectional** | **18-59** | **82.5/17.5** | **mixed** | **ELISA** | **A** |
| **Hassuna(2014)**[32] | **May 2011 -December 2011** | **5410** | **48** | **Minia** | **healthy blood donors** | **cross-sectional** | **19-58** | **79.6/20.4** | **31.7/68.3** | **ELISA** | **A** |
| **Habil(2013)**[33] | **Nov 2006 to Oct 2007** | **12000** | **237** | **Minia** | **Blood donors** | **cross-sectional** | **16-40** | **84/16** | **N/P** | **ELISA** | **A** |
| **Hussein(2014)**[34] | **2006 - 2012** | **308762** | **3756** | **Cairo** | **Blood donors** | **cross-sectional** | **28 ± 7.1 years** | **N/P** | **N/P** | **ELISA** | **B** |
| **Masoud (2020)**[35] | **2013-2014** | **11604** | **295** | **Qena** | **blood donors** | **cross-sectional** | **18-60** | **88.2/11.8** | **30.7/69.3** | **ELISA** | **A** |
| **Ismail(2009)**[36] | **2000-2007** | **55922** | **724** | **Mansoura** | **blood donors** | **cross sectional** | **30.98 ± 8.6** | **93.5/6.5** | **30/70** | **ELISA** | **A** |
| **Wasfi(2011)**[37] | **2007 to March 2008** | **3420** | **47** | **Alexandria** | **blood donors** | **cross-sectional** | **18-60** | **93.6/6.4** | **80.9/19.1** | **ELISA** | **A** |
| **Khattab(2010)**[38] | **2000 to 2008** | **211772** | **3507** | **Minia** | **blood donors** | **cross-sectional** | **N/P** | **85.9/14.1** | **63/37** | **ELISA** | **A** |
| **Lelie(2016)**[39] | **between 2005 and 2011** | **80631** | **849** | **Giza** | **blood donors** | **cross-sectional** | **N/P** | **N/P** | **N/P** | **chemoleucent- Assay** | **B** |
| **Hussein(2012)**[40] | **2011** | **3425** | **42** | **Cairo** | **blood donors** | **Comparative study** | **31.7±10.4** | **57.98/40.83** | **N N/P** | **ELISA** | **B** |
| **El-Gilany(2006)**[41] | **2002–2003** | **2157** | **93** | **Mansoura University** | **student voluntary blood donors** | **Retrospective cohort** | **17–24** | **62.6/37.4** | **63.1/36.9** | **ELISA** | **A** |
| **Abdel Messih(2014)**[42] | **August 2010 to January 2011** | **15017** | **265** | **Cairo** | **family replacement donors** | **case control** | **35** | **82.3/17.7** | **N/P** | **ELISA** | **A** |
| **Abdel Messih(2014)**[42] | **August 2010 to January 2011** | **2101** | **5** | **Cairo** | **voluntary donors** | **Cross-sectional** | **25** | **73.7/26.3** | **N/P** | **ELISA** | **A** |

*** Age± Standard deviation or age range
N/P: Not reported
M/F: Male/Female
U/R: urban/rural
ELISA: enzyme-linked immunosorbent assay**

**Table S4. Studies reporting hepatitis B virus (HBV) seroprevalence among populations at intermediate risk in Egypt.**

| **A:Health care workers( HCWs)(n=7)** | | | | | | | | | | |  |
| --- | --- | --- | --- | --- | --- | --- | --- | --- | --- | --- | --- |
| **First author last name, year of**  **publication [citation]** | **Duration of sample collection** | **Sample**  **size** | **HBs Ag positive patients** | **region** | **population** | **Study design** | **Age*** | **M/F (%)** | **U/R (%)** | **Method** | **quality** |
| **Anwar(2017)**[43] | **March to July 2016** | **175** | **0** | **Beni-Suef** | **HCWs** | **cross-sectional** | **20- > 50** | **12.6/87.4** | **53.9/46.1** | **Elisa** | **A** |
| **El-Melligy(2016)**[44] | **2013-2013** | **228** | **2** | **Cairo** | **HCWs** | **cross-sectional** | **<40 - ≥ 40** | **N/P** | **N/P** | **Elisa** | **B** |
| **Elmaghloub (2017)**[45] | **June 2014 and April 2015** | **564** | **8** | **Tanta** | **HCWs** | **Cross-sectional study** | **16-64** | **56.6/43.44** | **N/P** | **ELISA** | **A** |
| **Abdel Rasoul (2010)**[46] | N/P | **100** | **0** | **Menoufia** | **HCWs** | **cross-sectional** | **35.40±6.63*** | **27/73** | **60/40** | **ELISA** | **A** |
| **Zayet (2015)**[47] | N/P | **194** | **6** | **Assiut** | **HCWs** | **Cross-sectional** | **20- 40** | **56.3/43.7** | **N/P** | **chemoleucent- Assay** | **A** |
| **Aly (2020)**[48] | N/P | **450** | **2** | Suez | HCWs | **Cross-sectional** | 18-68 Y | 36.9%/63.1% | 100% urban | ELISA | **A** |
| **Abdelwahab(2012)**[49] | August 2008 to January 2010 | **842** | **13** | **Menoufia** | **HCWs** | **Cross-sectional** | **18-59** |  |  | **ELISA** | **B** |
| **B: others(n=3)** | | | | | | | | | | |  |
| **Hassanein(2019)**[50] | **April to Sep 2016** | **410** | **12** | **Alexandria** | **sewage  workers** | **cross-sectional** | **20-60** | **N/P** | **58.5/41.5** | **Elisa** | **A** |
| **Abd El-Wahab (2019)**[51] | **2018** | **1476** | **22** | **Alexandria** | **Waste  workers** | **cross-sectional** | **39.8 ±7.9** | **All males** | **1074(72.8%) urban, 402 (27.2%) Rural** | **ELISA** | **A** |
| **shalaby(2010)**[52] | **2007** | **616** | **25** | **Gharbeya** | **barbers and their clients** | **Cross-sectional** | **20–40** | **Male only** | **52.3/47.7** | **ELISA** | **A** |

*** Age± Standard deviation or age range**

**N/P: Not reported**

**M/F: Male/Female**

**U/R: urban/rural**

**ELISA: enzyme-linked immunosorbent assay**

**Table S5 Studies reporting hepatitis B virus (HBV) seroprevalence among populations at high risk in Egypt**

| **A:Patients with hemolytic anemia requiring blood transfusion or with end stage renal failure requiring hemodialysis (n=10)** | | | | | | | | | | |  |
| --- | --- | --- | --- | --- | --- | --- | --- | --- | --- | --- | --- |
| **First author last name, year of**  **publication [citation]** | **Duration of sample collection** | **Sample**  **size** | **HBs Ag positive patients** | **region** | **population** | **Study design** | **Age *** | **M/F (%)** | **U/R (%)** | **Method** | **Quality** |
| **Omar(2011)**[53] | **N/P** | **174** | **0** | **Cairo** | **thalassemic patients** | **cross-sectional** | **2 to 27** | **N/P** | **N/P** | **ELISA** | **B** |
| **Salama(2015)**[54] | **N/P** | **80** | **0** | **Cairo** | **thalassemic children** | **cross-sectional** | **5–18** | **53.7/46.3** | **N/P** | **ELISA** | **A** |
| **Mahmoud(2016)**[55] | **May 2014- May 2015** | **97** | **4** | **Sohag and Minia** | **thalassemic children** | **cross sectional** | **6–18** | **63.92/36.08** | **N/P** | **ELFA** | **B** |
| **El Faramawy(2012)**[56] | **N/P** | **100** | **12** | **Qena** | **multitransfused children** | **cross-sectional** | **4-15** | **68/32** | **N/P** | **ELISA** | **A** |
| **Abdel-Maksoud(2019)**[57] | **2015- 2016** | **150** | **17** | **Ismailia** | **hemodialysis patients** | **Prospective cohort** | **10-70** | **52.7/47.3** | **N/P** | **ELISA** | **A** |
| **Abd-Allah(2010)**[58] | **2007-2009** | **340** | **38** | **Cairo** | **hemodialysis patients** | **cross-sectional** | **50.9±11.6 years** | **78.2/21.8** | **N/P** | **ELISA** | **A** |
| **Mansour(2012)**[11] | **2009-2010** | **200** | **75** | **Mansoura** | **thalassemic patients** | **case control** | **11M-19Y** | **55.5/44.5** | **N/P** | **ELISA** | **A** |
| **Roshdy(2013)**[12] | **2010** | **40** | **3** | **Alexandria** | **thalasemic and AML** | **case control** | **20** | **N/P** | **N/P** | **ELISA** | **A** |
| **Elghannam(2009)**[16] | **N/P** | **143** | **4** | **Mansoura** | **hemodialysis patients** | **case control** | **N/P** | **66.5/33.5** | **N/P** | **ELISA** | **A** |
| **Bedewy(2006)**[17] | **N/P** | **116** | **0** | **Beheira** | **hemodialysis patients** | **case control** | **47.1 ± 14.8** | **N/P** | **N/P** | **ELISA** | **A** |
| **B:population with direct contact with HBV infected patients(n=1)** | | | | | | | | | | |  |
| **El-Sayed(2010)**[59] | **N/P** | **154** | **2** | **Cairo** | **household contacts of patients**  **with HBV**  **.** | **cross-sectional** | **N/P** | **N/P** | **N/P** | **ELISA** | **B** |
| **C: HIV patient infected patients(n=1)** | | | | | | | | | | |  |
| **Gumie(2019)**[60] | **2018-2019** | **141** | **9** | **Alexandria** | **HIV patient** | **Cross-sectional** | **<30 ->40** | **78.7/21.3** | **N/P** | **ELISA** | **B** |

*** Age± Standard deviation or age range**

**N/P: Not reported**

**M/F: Male/Female**

**U/R: urban/rural**

**ELISA: enzyme-linked immunosorbent assay**

**ELFA:** **Enzyme-linked fluorescence assay**

**Table S6 Studies reporting hepatitis B virus (HBV) seroprevalence patients with liver related conditions**

| **First author last name, year of**  **publication [citation]** | **Duration of sample collection** | **Sample**  **size** | **HBs Ag positive patients** | **region** | **population** | **Study design** | **Age*** | **M/F (%)** | **U/R (%)** | **Method** | **Quality** |
| --- | --- | --- | --- | --- | --- | --- | --- | --- | --- | --- | --- |
| **A:patients chronically infected with HCV** | | | | | | | | | | |  |
| **Ibrahim(2018)**[61] | **2015-2016** | **184** | **3** | **Kafer El-Sheikh  Gharbeya** | **patients chronically infected with HCV** | **cross-sectional** | **27-56** | **54.3/45.7** | **22.8/77.2** | **ELISA** | **B** |
| **Khedr(2020)**[10] | **N/P** | **124** | **1** | **N/P** | **HCV** | **case control** | **52.137±6.9** | **N/P** | **N/P** | **ELISA** | **B** |
| **B:patients with HCC** | | | | | | | | | | |  |
| **El-Zayadi(2005)**[62] | **1993-2002** | **1328** | **377** | **Cairo** | **HCC** | **Prospective**  **cohort** | **33 to 74** | **77.7/22.3** | **24.8/75.2** | **ELISA** | **A** |
| **Amer(2013)**[63] | **2007-2009** | **1034** | **108** | **Cairo, Menoufia, Zagazig** | **HCC** | **cross-sectional** | **30 to 76** | **78.5/21.5** | **N/P** | **ELISA** | **A** |
| **C:Non cirrhotic liver disease** | | | | | | | | | | |  |
| **El-Zayadi(2005)**[62] | **1993-2002** | **21122** | **4209** | **Cairo** | **Chronic liver disease** | **Prospective**  **cohort** | **33 to 74** | **77.7/22.3** | **24.8/75.2** | **ELISA** | **A** |
| **Amer(2013)**[63] | **2007-2009** | **93** | **47** | **Cairo, Menoufia, Zagazig** | **Chronic liver disease** | **cross-sectional** | **30 to 76** | **78.5/21.5** | **N/P** | **ELISA** | **A** |

*** Age± Standard deviation or age range**

**N/P: Not reported**

**M/F: Male/Female**

**U/R: urban/rural**

**ELISA: enzyme-linked immunosorbent assay**

**Table S7 Studies reporting hepatitis B virus (HBV) seroprevalence patients with special condition**

| **A:patients with malignancy(n=6)** | | | | | | | | | | |  |
| --- | --- | --- | --- | --- | --- | --- | --- | --- | --- | --- | --- |
| **First author last name, year of**  **publication [citation]** | **Duration of sample collection** | **Sample**  **size** | **HBs Ag positive patients** | **region** | **population** | **Study design** | **Age*** | **M/F (%)** | **U/R (%)** | **Method** | **Quality** |
| **Högfeldt(2016)**[64] | **2003-2008** | **47** | **18** | **Cairo** | **Hodgkin's lymphoma (NHL)** | **case control** | **N/P** | **N/P** | **N/P** | **chemoleucent- Assay** | **A** |
| **Sharaf-Eldeen(2007)**[65] | **N/P** | **100** | **9** | **Damietta  and Cairo** | **Children with Malignancy** | **cross-sectional** | **0.5 -14** | **61/39** | **N/P** | **ELISA** | **A** |
| **Said(2009)**[5] | **6-month period** | **100** | **45** | **Cairo** | **children with hematological disorders and malignancies** | **case control** | **11.4 ± 5.1** | **N/P** | **N/P** | **ELISA** | **A** |
| **El-Sayed(2006)**[15] | **2002** | **29** | **2** | **Cairo** | **Non-Hodgkin’s lymphoma** | **case control** | **14 (3.5-67)** | **N/P** | **N/P** | **ELISA** | **B** |
| **Nour El-deen(2011)**[14] | **N/P** | **40** | **13** | **Alexandria** | **AML patients with and without septicemia** | **case control** | **N/P** | **N/P** | **N/P** | **ELISA** | **A** |
| **Mostafa(2003)**[66] | **2000-2001** | **210** | **55** | **Cairo** | **pediatric malignancies** | **Prospective cohort** | **1-17** | **N/P** | **N/P** | **ELISA** | **B** |
| **B:heterogenous cases(n=4)** | | | | | | | | | | |  |
| **First author last name, year of**  **publication [citation]** | **Duration of sample collection** | **Sample**  **size** | **HBs Ag positive patients** | **region** | **population** | **Study design** | **Age range** | **M/F (%)** | **U/R (%)** | **Method** | **Quality** |
| **Abdel-metaal (2003)** [67] | **N/P** | **100** | **2** | **Assiut** | **rheumatoid arthritis patients** | **cross-sectional** | **9–71** | **14/86** | **N/P** | **ELISA** | **B** |
| **Abdel-Noor( 2019)** [68] | **N/P** | **102** | **2** | **Tanta** | **Rheumatic disease patients** | **cross sectional** | **17-70** | **3.9/96.1** | **mixed** | **ELISA** | **B** |
| **Kandil(2007)**[13] | **2004-2006** | **80** | **3** | **Cairo** | **Type 1 DM, Chronic renal failure and SLE** | **case control** | **12.06±3.84** | **N/P** | **N/P** | **ELISA** | **A** |
| **Elrashidy(2013)**[6] | **2012-2013** | **63** | **0** | **Cairo** | **insulin-dependent diabetes mellitus (IDDM)** | **case control** | **4–17** | **N/P** | **N/P** | **ELISA** | **B** |

*** Age± Standard deviation or age range**

**N/P: Not reported**

**M/F: Male/Female**

**U/R: urban/rural**

**ELISA: enzyme-linked immunosorbent assay**

**AML:** **acute myeloid leukemia**

**Table S8 Prevalence of** **unprotective levels of anti-HBs (< 10 IU/L) in population with a history of vaccination during infancy**

| **first author(publication time)** | **study period** | **sample size** | **HBs Ag positive patients** | **population** | **anti-HBs < 10 IU/L by age group*** | | | |
| --- | --- | --- | --- | --- | --- | --- | --- | --- |
|  |  |  |  |  | **less than 5 years** | **5-10y** | **10-15 Y** | **>15Y** |
| **Abushady(2011)**[7] | **2004 to January 2007** | **600*** | **5** | **healthy** **Children** | **56/200(28%)** |  |  |  |
| **Salama(2015)**[1] | **July 2010 to June 2013** | **3600** | **4** | **healthy Children** | **152/1114(13.6%)** | **206/625(33%)** | **606/1026(59%)** | **571/821(69.5%)** |
| **Kishk(2020)**[21] | **January 2018 to January 2019** | **600** | **30** | **pregnant** |  |  |  | **131/285(46%)** |
| **Sami (2016)**[3] | **July 2010 to June 2013** | **189** | **0** | **children** | **10/189(5.3%)** | **16/189(8.5%)** | **62/189(32.8)** |  |
| **El-Sayed(2021)**[19] | **September 2015 to September 2017.** | **400** | **29** | **university students** |  |  |  | **218/400(54.5%)** |

***number of participants that have unprotective levels of anti-HBs (less than 10 IU/L) divided by the total number of participants in each age category.**

**Table S9 seroprevalence of HBV according to gender**

| **First author(publication time)(citation)** | **study period** | **sample size** | **population** | **Male(n)** | **HBsAg +(n)** | **female (n)** | **HBsAg +** (**n)** |
| --- | --- | --- | --- | --- | --- | --- | --- |
| **El-Zayadi(2008)**[30] | **2005** | **760** | **blood donors** | **636** | **8** | **124** | **1** |
| **Awadalla(2011)**[31] |  | **1000** | **blood donors** | **825** | **45** | **175** | **5** |
| **Hassuna(2014)**[32] | **May 2011 till December 2011** | **5410** | **blood donors** | **4305** | **43** | **1105** | **5** |
| **Masoud(2020)**[35] | **Jan. 2013 to Jan.2014** | **11604** | **blood donors** | **10232** | **278** | **1372** | **17** |
| **El-Gilany(2006)**[41] | **2002–03** | **2157** | **blood donors** | **1350** | **69** | **807** | **24** |
| **Soliman(2019)**[68] | **June 2016 and May 2017** | **67,042** | **healthy** | **31945** | **1982** | **35062** | **965** |
| **El-Faramawy(2012)** |  | **100** | **Multitransfused patients.** | **68** | **8** | **32** | **4** |
| **Ibrahim(2018)**[61] | **March 2015 and April 2016** | **184** | **HCV infected patients** | **100** | **1** | **84** | **2** |
| **Mahmoud(2016)**[55] | **May 2014 to May 2015** | **97** | **Multitransfused Thalassemic Children** | **62** | **3** | **35** | **1** |

**Table S10 seroprevalence of HBV according to setting**

| **First author(publication time)(citation)** | **study period** | **sample size** | **population** | **urban (n)** | **HBsAg +(n)** | **rural (n)** | **HBsAg +(n)** |
| --- | --- | --- | --- | --- | --- | --- | --- |
| **Hassuna(2014)**[32] | **May 2011 till December 2011** | **5410** | **blood donors** | **1715** | **15** | **3695** | **33** |
| **Masoud(2020)**[35] | **Jan. 2013 to Jan.2014** | **11604** | **blood donors** | **3564** | **53** | **8040** | **242** |
| **Zayet (2015)**[47] |  | **215** |  | **322** | **10** | **294** | **3** |
| **shalaby(2010)**[52] | **2007** | **616** | **healthy** | **308** | **19** | **308** | **6** |
| **El-Gilany(2006)**[41] | **2002–03** | **2157** | **blood donors** | **1362** | **52** | **795** | **41** |
| **Abo-Salem(2014)**[27] | **N/P** | **397** | **pregnant women** | **140** | **4** | **257** | **5** |
| **Ibrahim(2018)**[61] | **March 2015 and April 2016** | **184** | **HCV infected patients** | **42** | **0** | **142** | **3** |
| **Abd El-Wahab(2019)**[51] | **2/1/2018** | **1476** | **Municipal sewage workers** | **1074** | **17** | **402** | **5** |

**
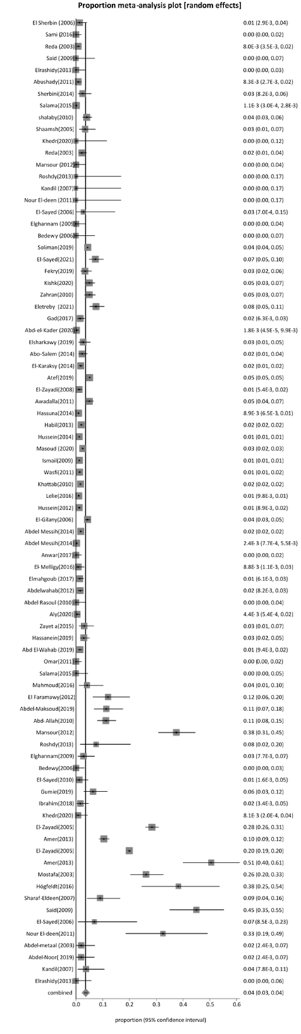
Fig. S1**

**Forest plot of the prevalence of hepatitis B virus infection in all subpopulations in Egypt.**

**Fig. S2**

Forest plot of HBV prevalence among male participants ****

**Fig. S3**

Forest plot of HBV prevalence among female participants

**Fig. S4**

Forest plot of HBV prevalence in urban areas in Egypt

**Fig. S5
**

Forest plot of HBV prevalence in rural areas in Egypt

**References:**

1. Salama II, Sami SM, Said ZNA, et al. Effectiveness of hepatitis B virus vaccination program in Egypt: Multicenter national project. *World J Hepatol* 2015;7(22):2418. doi:10.4254/WJH.V7.I22.2418

2. El Sherbini A, Mohsen SA, Seleem Z, Ghany AA, Moneib A, Abaza AH. Hepatitis B virus among schoolchildren in an endemic area in Egypt over a decade: Impact of hepatitis B vaccine. *Am J Infect Control* 2006;34(9):600-602. doi:10.1016/j.ajic.2005.12.018

3. Sami SM, Salama II, Abdel-Latif GA, El Etreby LA, Metwally AI, El Haliem NFA. Hepatitis B Seroprotection and the Response to a Challenging Dose among Vaccinated Children in Red Sea Governorate. *Open Access Maced J Med Sci* 2016;4(2):219. doi:10.3889/OAMJMS.2016.043

4. Reda AA, Arafa MA, Youssry AA, Wandan EH, Ab de Ati M, Daebees H. Epidemiologic evaluation of the immunity against hepatitis B in Alexandria, Egypt. *Eur J Epidemiol 2003 1810* 2003;18(10):1007-1011. doi:10.1023/A:1025805817101

5. Said ZNA, El-Sayed MH, El-Bishbishi IA, et al. High prevalence of occult hepatitis B in hepatitis C-infected Egyptian children with haematological disorders and malignancies. *Liver Int* 2009;29(4):518-524. doi:10.1111/J.1478-3231.2009.01975.X

6. Elrashidy H, Elbahrawy A, El-Didamony G, et al. Antibody levels against hepatitis B virus after hepatitis B vaccination in Egyptian diabetic children and adolescents. *Hum Vaccines Immunother* 2013;9(9):2002-2006. doi:10.4161/hv.25426

7. Abushady EAE, Gameel MMA, Klena JD, Ahmed SF, Abdel-Wahab KSE, Fahmy SM. HBV vaccine efficacy and detection and genotyping of vaccineé asymptomatic breakthrough HBV infection in Egypt. *World J Hepatol* 2011;3(6):147. doi:10.4254/WJH.V3.I6.147

8. Sherbini AS, Sherbiny HS. Immunoprophylaxis of Compulsory Hepatitis B Vaccination in Sharkia, Egypt. *Afro-Egyptian J Infect Endem Dis* 2014;4(1):23-31. doi:10.21608/AEJI.2014.16445

9. Shaamsh AH, Salem HT, Shaaban MM, Ghaneima SA, Helal SR. Effect of uniplant on liver function in Egyptian women with asymptomatic hepatitis B virus infection. *Afr J Reprod Health* 2005;9(1):24-31. doi:10.2307/3583157

10. Khedr A. Incidence and Clinical Implications of Isolated Hepatitis B Core Antibody Serologic Profile Pattern Among Egyptian Patients with Chronic Hepatitis C. *Egypt Acad J Biol Sci C, Physiol Mol Biol* 2020;12(2):169-180. doi:10.21608/eajbsc.2020.133909

11. Mansour AK, Aly RM, Abdelrazek SY, et al. Prevalence of HBV and HCV infection among multi-transfused Egyptian thalassemic patients. *Hematol Oncol Stem Cell Ther* 2012;5(1):54-59. doi:10.5144/1658-3876.2012.54

12. Roshdy MN, Harfoush RA, Hamed NA, Morsi MG. Quantitative estimation of interferon-gamma levels among Egyptian polytransfused haematology cases. *East Mediterr Heal J* 2013;19(5):490-494. doi:10.26719/2013.19.5.490

13. Kandil ME, Rasheed MA, Saad NE. Hepatitis C and B viruses among some high risk groups of Egyptian children. *J Med Sci* 2007;7(8):1259-1267. doi:10.3923/JMS.2007.1259.1267

14. el deen RAA, Harfoush RA, Elgharabawy MM, Hamed NA, Morsi MG. Levels of interleukins 12 (IL-12) and 13 (IL-13), hepatitis B and C serology, and blood cultures among acute myeloid leukemia (AML) patients in Egypt. *J Venom Anim Toxins Incl Trop Dis* 2011;17(3):293-299. doi:10.1590/s1678-91992011000300009

15. El-Sayed GM, Mohamed WSE din, Nouh MA, Moneer MM, El-Mahallawy HA. Viral genomes and antigen detection of hepatitis B and C viruses in involved lymph nodes of Egyptian non-Hodgkin’s lymphoma patients. The Egyptian journal of immunology / Egyptian Association of Immunologists. Published 2006. Accessed August 28, 2022. https://pubmed.ncbi.nlm.nih.gov/17974155/

16. Elghannam D, Aly R, Goda E, Eltoraby E, Farag R. Clinical significance of antibody to hepatitis B core antigen in multitransfused hemodialysis patients. *Asian J Transfus Sci* 2009;3(1):14. doi:10.4103/0973-6247.45256

17. Bedewy K, Yousry NI. TT-Virus and Occult Hepatitis B Virus Infections in Egyptian. *Egypt J Med Microbiol* 2006;15(1).

18. Soliman G, Elzalabany M, Hassanein T, Miller D. Mass screening for hepatitis B and C in South Upper Egypt: lessons learned from a real life experience. *J Hepatol* 2018;68:S180-S181. doi:10.1016/S0168-8278(18)30573-7

19. El-Sayed NA, Elshazly SH, Said ZN, Ela AM. El, Abdelmageed NA. Hepatitis B Seroprevalence among Egyptian University students in the postinfant compulsory vaccination period. *Sci J Al-Azhar Med Fac Girls* 2021;5(1):84. doi:10.4103/SJAMF.SJAMF_31_21

20. Fekry MM, Hashish MH, Selim HS, Fawzy AM, Wahba MM. Prevalence of Hepatitis B Virus among Pregnant Women Attending Antenatal Care in Alexandria. *J High Inst Public Heal* 2019;49(3):175-179. doi:10.21608/JHIPH.2019.63795

21. Kishk R, Mandour M, Elprince M, et al. Pattern and interpretation of hepatitis B virus markers among pregnant women in North East Egypt. *Brazilian J Microbiol* 2020;51(2):593-600. doi:10.1007/S42770-019-00174-3/FIGURES/1

22. Zahran KM, Badary MS, Agban MN, Abdel Aziz NHR. Pattern of hepatitis virus infection among pregnant women and their newborns at the Women’s Health Center of Assiut University, Upper Egypt. *Int J Gynaecol Obstet* 2010;111(2):171-174. doi:10.1016/J.IJGO.2010.06.013

23. Eletreby R, Elraouf MA, Fouad A, et al. Screening for chronic hepatitis C and chronic hepatitis B infections among pregnant females: a cross-sectional study. *Egypt Liver J* 2021;11(1). doi:10.1186/s43066-021-00113-8

24. Gad MA, Metwally MA, Eissa HA, Gehad MA, Rayan MM. Antenatal screening for hepatitis B virus infection. *Benha Med J* 2017;34(2):113. doi:10.4103/1110-208X.218829

25. Abdelkader AH, Ibrahim SA. Prevalence of Hepatitis B and C Virus Infection among Pregnant Women in Sharkia Governorate, Egypt. *Afro-Egypt J Infect Endem Dis* 2020;10(2):200-206. https://aeji.journals.ekb.eg/http://mis.zu.edu.eg/ajied/home.aspx

26. Elsharkawy SS, Elgazayerli WS, Elsharkawy SS, Elgazayerli WS. Sero-Prevalence of HBV, HCV and HEV among the Egyptian Pregnant Females. *Open J Obstet Gynecol* 2017;9:1429-1438. doi:10.4236/ojog.2019.910138

27. Abo-Salem MES, Mahrous OAE, El-Shaarawy AA, Mohamed HM, Yehia SAS. Seroprevalence of hepatitis B among pregnant women attending maternal and child health centres in Shebin El-Kom district (Menoufia governorate). *Menoufia Med J* 2014;27(4):847. doi:10.4103/1110-2098.149805

28. El-Karaksy HM, Mohsen LM, Saleh DA, et al. Applicability and efficacy of a model for prevention of perinatal transmission of hepatitis B virus infection: Single center study in Egypt. *World J Gastroenterol* 2014;20(45):17075. doi:10.3748/WJG.V20.I45.17075

29. Atef DM, Atef RM. Usefulness of nucleic acid testing among negative HBs Ag blood donors in Egypt. *Transfus Apher Sci* 2019;58(4):468-471. doi:10.1016/J.TRANSCI.2019.05.005

30. El-Zayadi AR, Ibrahim EH, Badran HM, et al. Anti-HBc screening in Egyptian blood donors reduces the risk of hepatitis B virus transmission. *Transfus Med* 2008;18(1):55-61. doi:10.1111/J.1365-3148.2007.00806.X

31. Awadalla HI, Ragab MH, Osman MA, Nassar NA. Risk Factors of Viral Hepatitis B among Egyptian Blood Donors. *Br J Med Med Res* Published online 2011. Accessed August 28, 2022. http://imsear.searo.who.int/handle/123456789/162602

32. Hassuna NA, Mohamed ZM, Abo-Eleuoon M, Abdel-Hamid M, Xu J. Prevalence of Hepatitis B Virus (HBV), Hepatitis C Virus (HCV) Infections and their Co-infection among Blood Donors in Minia Governorate, Egypt. *J Adv Med Med Res* 2015;5(8):987-993. doi:10.9734/BJMMR/2015/12901

33. Habil FE, Mahdi WKM, Abdelwahab SF, Abdel-Hamid M. Hepatitis B virus genotype D predominates HBsAg-positive Egyptian blood donors and is mainly associated with a negative HBeAg serostatus. *Intervirology* 2013;56(5):278-283. doi:10.1159/000353105

34. Hussein E. Blood donor recruitment strategies and their impact on blood safety in Egypt. *Transfus Apher Sci* 2014;50(1):63-67. doi:10.1016/J.TRANSCI.2013.11.005

35. Masoud A, Ahmed A, Temerk HA, et al. The seropervelance of infectious hepatitis viruses (HBV, HCV and HEV) among blood donors and their correlation to risk factors in Qena governorate, Upper Egypt. *VirusDisease* 31. doi:10.1007/s13337-020-00589-9

36. Ismail AM, Ziada HN, Sheashaa HA, Shehab El-Din AB. Decline of viral hepatitis prevalence among asymptomatic Egyptian blood donors: A glimmer of hope. *Eur J Intern Med* 2009;20(5):490-493. doi:10.1016/J.EJIM.2009.03.005

37. Wasfi OAS, Sadek NA. Prevalence of hepatitis B surface antigen and hepatitis C virus antibodies among blood donors in Alexandria, Egypt. Eastern Mediterranean Health Journal. doi:10.26719/2011.17.3.238

38. Khattab MA, Eslam M, Sharwae MA, Hamdy L. Seroprevalence of hepatitis C and B among blood donors in Egypt: Minya Governorate, 2000-2008. *Am J Infect Control* 2010;38(8):640-641. doi:10.1016/j.ajic.2009.12.016

39. Lelie N, Bruhn R, Busch M, et al. Detection of different categories of hepatitis B virus (HBV) infection in a multi-regional study comparing the clinical sensitivity of hepatitis B surface antigen and HBV-DNA testing. *Transfusion* 2017;57(1):24-35. doi:10.1111/TRF.13819

40. Hussein E, Teruya J. Evaluation of blood supply operation and infectious disease markers in blood donors during the Egyptian revolution. *Transfusion* 2012;52(11):2321-2328. doi:10.1111/J.1537-2995.2012.03592.X

41. El-Gilany AH, El-Fedawy S. Bloodborne infections among student voluntary blood donors in Mansoura University, Egypt. Eastern Mediterranean Health Journal. Published 2006. Accessed August 28, 2022. https://pubmed.ncbi.nlm.nih.gov/17333818/

42. Abdel Messih IY, Ismail MA, Saad AA, Azer MR. The degree of safety of family replacement donors versus voluntary non-remunerated donors in an Egyptian population: a comparative study. *Blood Transfus* 2014;12(2):159. doi:10.2450/2012.0115-12

43. Anwar MM, Ahmed D, Sheemy M, El-Tayeb M. Seroprevalence and risk factors for Hepatitis B and C among health care workers. *Int J Infect Control* 2017;13(2):2. doi:10.3396/IJIC.V13I2.17459

44. El-Melligy DM, Saad-Hussein A, Khalil SA. Occupational exposure to hepatitis infection among Egyptian healthcare workers and hepatitis B vaccination. *J Arab Soc Med Res* 2016;11(1):14. doi:10.4103/1687-4293.186777

45. Elmaghloub R, Elbahrawy A, Didamony G El, et al. Hepatitis B virus genotype E infection among Egyptian health care workers. *J Transl Intern Med* 2017;5(2):100-105. doi:10.1515/JTIM-2017-0012

46. Abdel Rasoul G, El Bahnasy R, Michael A, Hendy O, Ahmed A. Hepatitis B Viral Markers and Vaccination Status Among Health Care Providers in Menoufia Governorate. Egypt J Occup Med 2010;34(2):267-279. doi:10.21608/ejom.2010.726

47. Zayet H, Ezz El-Din A, Ahmed S, El-Khayat M. Hepatitis B and C Virus Infection Among Health Care Workers in General Surgery Department, Assiut Univers Ity Hospitals. Egypt J Occup Med 2015;39(1):85-104. doi:10.21608/ejom.2015.813

48. Aly H, Soliman N, Nemr N, et al. Hepatitis B Virus Sero-prevalence and Vaccination Status among Health Care Workers, North East Egypt. *Egypt J Med Microbiol* 2020;29(2):169-177. doi:10.21608/EJMM.2020.250262

49. Abdelwahab S, Rewisha E, Hashem M, et al. Risk factors for hepatitis C virus infection among Egyptian healthcare workers in a national liver diseases referral centre. *Trans R Soc Trop Med Hyg* 2012;106(2):98-103. doi:10.1016/j.trstmh.2011.10.003

50. Hassanein F, Masoud I, Shehata A. Infection hazard of exposure to intestinal parasites, H. pylori and hepatitis viruses among municipal sewage workers: a neglect high risk population. *Parasitol United J* 2019;12(2):130-138. doi:10.21608/PUJ.2019.13679.1047

51. Abd El-Wahab EW, Eassa SM. Seroprevalence of HBV among Egyptian municipal solid waste workers. *Heliyon* 2019;5(6):e01873. doi:10.1016/j.heliyon.2019.e01873

52. Shalaby S, Kabbash IA, El Saleet G, Mansour N, Omar A, El Nawawy A. Hepatitis B and C viral infection: Prevalence, knowledge, attitude and practice among barbers and clients in Gharbia governorate, Egypt (Eastern Mediterranean Health Journal (2010), 16, 1, (10-17)). Eastern Mediterranean Health Journal. Published 2010. Accessed August 28, 2022. https://pubmed.ncbi.nlm.nih.gov/20214151/

53. Omar N, Salama K, Adolf S, El-Saeed GSM, Abdel Ghaffar N, Ezzat N. Major risk of blood transfusion in hemolytic anemia patients. *Blood Coagul Fibrinolysis* 2011;22(4):280-284. doi:10.1097/MBC.0b013e3283451255

54. Salama K, Ibrahim O, Kaddah A. Liver Enzymes in Children with beta-Thalassemia Major: Correlation with Iron Overload and Viral Hepatitis. *Artic Open Access Maced J Med Sci* Published online 2015. doi:10.3889/oamjms.2015.059

55. Mahmoud RA, El-Mazary AAM, Khodeary A. Seroprevalence of Hepatitis C, Hepatitis B, Cytomegalovirus, and Human Immunodeficiency Viruses in Multitransfused Thalassemic Children in Upper Egypt. *Adv Hematol* 2016;2016. doi:10.1155/2016/9032627

56. El-Faramawy AAM, El-Rashidy OF, Tawfik PH, Hussein GH. Transfusion Transmitted Hepatitis: Where Do We Stand Now? A One Center Study in Upper Egypt. *Hepat Mon* 2012;12(4):286. doi:10.5812/HEPATMON.852

57. Abdel-Maksoud NHM, El-Shamy A, Fawzy M, Gomaa HHA, Eltarabilli MMA. Hepatitis B variants among Egyptian patients undergoing hemodialysis. *Microbiol Immunol* 2019;63(2):77-84. doi:10.1111/1348-0421.12670

58. Abd-Allah E, Waked E, Assal HS, Younes K, Kantoush N. Incidence of Hepatitis C Virus (HCV), Hepatitis B Virus (HBV) and Dual Infection in Egyptian Patients on Haemodialysis. *Kidney 2010 195* 2010;19(5):225-228. doi:10.1007/S00596-010-0144-8

59. Manal Hamdy ES, Naglaa Ahmed A, Marwa Gomaa EF. Intrafamilial transmission of hepatitis B and C among families of multi-transfused Egyptian children. *Egypt J Community Med* 2010;28(3):53-67. https://pesquisa.bvsalud.org/gim/resource/en/emr-135713

60. Gumie M, Saeed AB, Gad A, Abdelrahman AE, Elgayar A. Prevalence of Hepatitis C virus (HCV) and Hepatitis B Virus (HBV) Co-infection among Human Immunodeficiency Virus (HIV/AIDS). *Egypt J Hosp Med* 2019;77(1):4810-4814. doi:10.21608/EJHM.2019.47002

61. Ibrahim H, Ghaffar F, Shaer R, Madian M. Prevalence of Epstein-Barr Virus and Hepatitis B Virus Infections among Chronic HCV Patients Attending Kafer El Shiekh Liver and Heart Institute, Egypt. *J Adv Med Med Res* 2018;26(8):1-8. doi:10.9734/JAMMR/2018/41753

62. El-Zayadi AR, Badran HM, Barakat EMF, et al. Hepatocellular carcinoma in Egypt: A single center study over a decade. *World J Gastroenterol* 2005;11(33):5193. doi:10.3748/WJG.V11.I33.5193

63. Amer NA, Gemaay MA, Mohamed AE, Hussein MM, Shehad I. Prevalence of viral hepatitis in Egyptian patients with hepatocellular carcinoma. *Egypt Liver J* 2013;3(1):6-9. doi:10.1097/01.ELX.0000424247.25858.79

64. Högfeldt T, Jaing C, McLoughlin K, et al. Differential expression of viral agents in lymphoma tissues of patients with ABC diffuse large B-cell lymphoma from high and low endemic infectious disease regions. *Oncol Lett* 2016;12(4):2782-2788. doi:10.3892/ol.2016.5012

65. Sharaf-Eldeen S, Salama K, Eldemerdash S, Hassan HMS, Semesem M. Hepatitis B and C Viruses in Egyptian children with malignancy. *J Med Sci* 2007;7(6):1003-1008. doi:10.3923/JMS.2007.1003.1008

66. Mostafa A, Mansour T, Amin M, Khairy A, El Zomor H. Seroprevalence of Hepatitis B and C in Pediatric Malignanices. *J Egypt Nat Cancer Inst* 2003;15(1):33-42.

67. Gamal Eldin Abdel-metaal M, Abdulaal Doma M. PREVALENCE OF HEPATITIS B AND C IN RHEUMATOID ARTHRITIS. *AAMJ* 2003;1(2).

68. Abdel-Noor R, Watany M, Abd-Elsalam S, ElKhalawany W, Soliman S, Badawi R. Is Hepatitis B Surface Antigen (HB s Ag) Enough Alone as a Screening Test for HBV Infection in Rheumatic Disease Patients Before Starting Immunosuppressive Therapies? A Cross-sectional Study. *Infect Disord - Drug Targets* 2019;20(6):878-883. doi:10.2174/1871526519666191212094141

**Additional figures legends**

**Fig.S1: Forest plot of HBV seroprevalence in Egypt for studies published from 2000 to 2022**

**Fig.S2: Forest plot of HBV seroprevalence among males**

**Fig.S3: Forest plot of HBV seroprevalence among females**

**Fig.S4: Forest plot of HBV seroprevalence among urban settings**

**Fig.S5: Forest plot of HBV among rural settings**
